# Genome-wide identification of vegetative phase transition-associated microRNAs and target predictions using degradome sequencing in *Malus hupehensis*

**DOI:** 10.1186/1471-2164-15-1125

**Published:** 2014-12-17

**Authors:** Libo Xing, Dong Zhang, Youmei Li, Caiping Zhao, Songwen Zhang, Yawen Shen, Na An, Mingyu Han

**Affiliations:** College of Horticulture, Northwest A&F University, Yangling, Shaanxi 712100 China

**Keywords:** Phase change, Malus hupehensis, Small RNA, miRNA targets, Expression profile, Degradome

## Abstract

**Background:**

A long juvenile period between germination and flowering is a common characteristic among fruit trees, including *Malus hupehensis* (*Pamp.*) Rehd., which is an apple rootstock widely used in China. microRNAs (miRNAs) play an important role in the regulation of phase transition and reproductive growth processes.

**Results:**

*M. hupehensis* RNA libraries, one adult and one juvenile phase, were constructed using tree leaves and underwent high-throughput sequencing. We identified 42 known miRNA families and 172 novel miRNAs. We also identified 127 targets for 25 known miRNA families and 168 targets for 35 unique novel miRNAs using degradome sequencing. The identified miRNA targets were categorized into 58 biological processes, and the 123 targets of known miRNAs were associated with phase transition processes. The KEGG analysis revealed that these targets were involved in starch and sucrose metabolism, and plant hormone signal transduction. Expression profiling of miRNAs and their targets indicated multiple regulatory functions in the phase transition. The higher expression level of mdm-miR156 and lower expression level of mdm-miR172 in the juvenile phase leaves implied that these two small miRNAs regulated the phase transition. mdm-miR160 and miRNA393, which regulate genes involved in auxin signal transduction, could also be involved in controlling this process. The identification of known and novel miRNAs and their targets provides new information on this regulatory process in *M. hupehensis*, which will contribute to the understanding of miRNA functions during growth, phase transition and reproduction in woody fruit trees.

**Conclusions:**

The combination of sRNA and degradome sequencing can be used to better illustrate the profiling of hormone-regulated miRNAs and miRNA targets involving complex regulatory networks, which will contribute to the understanding of miRNA functions during growth, phase transition and reproductive growth in perennial woody fruit trees.

**Electronic supplementary material:**

The online version of this article (doi:10.1186/1471-2164-15-1125) contains supplementary material, which is available to authorized users.

## Background

Fruit trees, like most perennial woody plants, have a long juvenile phase before flowering and fruiting [1–3]. Studies of the mechanisms involved in juvenile development and the juvenile to adult phase transition are vital for shortening the juvenile phase and accelerating the breeding of economically beneficial traits in woody plants, especially in apple trees. Rootstock breeding is an important aspect of fruit breeding, since many apple trees are propagated by grafting a scion on a rootstock, such as T337 and M26. Shortening the rootstock’s juvenile phase is necessary for early flowering and fruiting of several scion varieties [4]. Presently, China produces more apples than any other country in the world and up to 90% of the rootstocks are bred using seed propagation [5]. *Malus hupehensis*, which originated in Pingyi, Shandong Province, is an important apple rootstock that undergoes apomixis at a rate greater than 95% [4].

Many species have long juvenile vegetative phases. This may be associated with the biological processes involved in hormone synthesis and metabolism, carbohydrate synthesis and metabolism, and photosynthesis [1, 6]. It has been reported that carbohydrates play an important role in the reproductive development of olive trees, [7] and that the *SQUAMOSA* promoter binding protein (SBP)-box genes *SPL10, SPL11* and *SPL2* control morphological changes in *Arabidopsis* reproduction [8]. Transgenic experiments in *Populus trichocarpa* and *Arabidopsis*, indicate that the terminal flower 1 (*TFL1*) gene expression level affects the transition from the vegetative to reproductive phase [9, 10]. Additionally, other genes, including the MADS-box family of transcription factor (TF) genes in citrus, are involved in the juvenile to adult transition [11]. These control mechanisms represent a preliminary understanding of the molecular base of the transition from juvenile to adult phase in plants.

MicroRNAs (miRNAs) play an important role in the regulating the juvenile to adult phase transition in annual plants [12, 13]. For example, in *Arabidopsis thaliana*, high levels of miR156 reduced the expression levels of SPL TFs, which activated *SUPPRESSOR of CONSTANS 1 (SOC1), LEAFY (LFY), AGAMOUS-LIKE 42 (AGL42)*, *FRUITFULL (FUL)* and *APETALA1 (AP1)* genes that regulate the transition from juvenile to adult phase [14]. miR156 acts in several pathways that control different aspects of vegetative development and play an important role in the juvenile phase [12]. miR172 down-regulates *GLOSSY15* expression, which promotes the vegetative phase change in maize [15]. In perennial woody species, related studies on the molecular mechanisms of phase change have been performed [16, 17]. The overexpression of miR156 in transgenic *Populus* × *canadensis* reduced the expression of miR156-targeted *SPL* genes and miR172, and drastically prolonged the juvenile phase [1]. The increase in SsmiR156 and decrease in SsmiR172 during plant rejuvenation showed that these two miRNAs affect phase transition in *Sequoia sempervirens*[18].

Plant hormones are known to play an important role in the juvenile to adult phase transition and in plant flowering [19]. Currently, the miRNA-mediated regulation of plant growth, development and flowering through phytohormone crosstalk and other developmental processes mainly involves six classes of phytohormones, auxin (AUX), cytokinin (CK), abscisic acid (ABA), gibberellic acid (GA), ethylene (ET) and jasmonic acid (JA) [20]. It was reported that GA accelerates flowering through the degradation of transcription repressors, DELLAs, and that DELLAs directly bind to miRNA156-targeted TFs (SPL family members), which promote flowering by activating miR172 and MADS-box genes [21].

We determine if miRNAs are involved in hormone regulation during the transition from vegetative to reproductive growth in apple trees. Additionally, we investigated the complex hormonal and miRNA-mediated regulatory networks in which the miRNAs associated with phase transition control plant growth, development and the transition to flowering.

## Methods

### Plant material and RNA isolation

**Figure 1:**
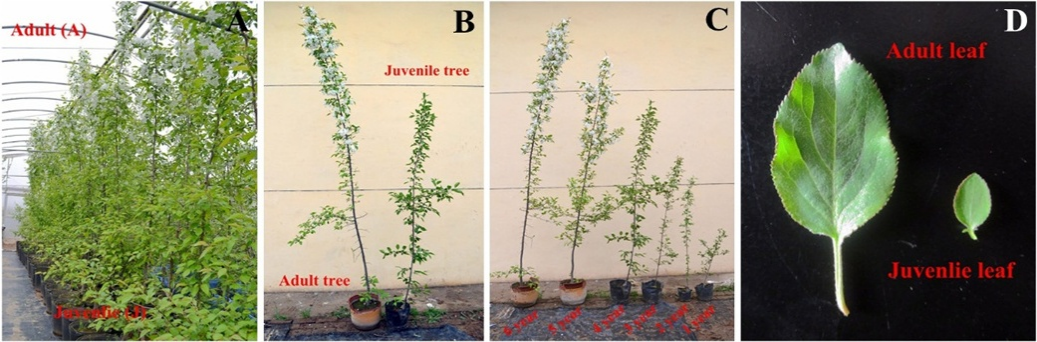
**Morphology of adult and juvenile trees in** ***Malus hupehensis*** **.** Adult phase at the tree top and juvenile phase at the tree base **(A)**; Adult tree that can flower and juvenile tree that cannot flower **(B)**; Trees of different ages; and **(C)** Adult leaves at the tree top and juvenile leaves at the tree base **(D)**.

Leaf samples from the Apple Demonstration Nursery of Yangling Modern Agriculture Technology Park (Northwest Agriculture & Forestry University), Shaanxi Province of China (34° 52′ N, 108° 7′ E), were collected directly into liquid nitrogen. In June, ‘Adult phase’ (A) leaves were collected as mixed samples from the tops of 18 6-year-old *M. hupehensis* (*Pamp.*) Rehd. trees and ‘Juvenile phase’ (J) leaves were collected from the base. Since *M. hupehensis* has the ability to undergo apomixis at a rate greater than 95%, their growth is highly synchronous. Roots, stems, flowers and fruits were also collected in the same manner at the same time. The samples were stored in a −80°C freezer until used (Figure 1). Additionally, leaf samples were collected from the tops of *M. hupehensis* of different ages (1-, 2-, 3-, 4-, 5- and 6-year-old trees) (Figure 1C). Two leaf samples, A and J, were used for small RNA and degradome sequencing, and the samples were used for qRT-PCR to verify the expression patterns of miRNA and their targets (Figure 1C,D). Total RNA was isolated from each sample by a modified method [22].

### Construction of small RNA and degradome libraries

Small RNA construction and deep sequencing of the A and J leaf samples was carried out by the Beijing Genomics Institute (BGI) (Shenzhen, China) using an Illumina Genome Analyzer [23]. Total RNA extractions from A and J leaves were performed using the RNeasyPlant Mini Kit (Qiagen, Hilden, Germany) and collected into A and J RNA pools. Briefly, 18–30-nt gel fragments were selected and then ligated to a pair of adapters at the 5′- and 3′-ends using T4 RNA ligase. The small RNAs with the adapters were transcribed into cDNA using Super-Script II Reverse Transcriptase (Invitrogen, Shanghai), and then the cDNA products were amplified by polymerase chain reaction (PCR). Finally, the purified PCR products were directly sequenced using Solexa sequencing technology (BGI). The leaf total RNA from the J sample was also used for degradome sequencing and library construction as previously described [24, 25], as well as for miRNA target identification.

### Bioinformatics analyses of sequencing data

Raw reads produced using an Illumina 1G Genome Analyzer at BGI were processed into clean full-length reads by removing the low-quality reads (e.g., those larger than 30 nt or smaller than 18 nt, those with between 30 and 50 adapter contaminants, those with polyA sequences and those without insert tags) by a data-cleaning pipeline. All high-quality sequences were queried using NCBI GenBank (http://www.ncbi.nlm.nih.gov/genbank) databases and Rfam databases (http://www.sanger.ac.uk/resources/databases/rfam.html), and used for further analyses. The small RNA tags were annotated with rRNA, scRNA, snoRNA, snRNA and tRNA using the tag2annotation software developed by BGI. To map every unique small RNA to only one annotation, we followed the following priority rule: rRNAetc (GenBank > Rfam) > known miRNA > repeat > exon > intron3 (Shenzhen, China).

### Identification of known miRNAs and prediction of novel miRNAs in *M. hupehensis (Pamp.)*Rehd

To identify known miRNAs in *M. hupehensis*, the miRNA categories were mapped to the reference genome *Malus domestica* in miRBase 18.0 (http://www.mirbase.org) with the criterion that sequences in the small RNA libraries (A and J) have less than two mismatches and more than 16 matches without gaps. miRNAs that could not be annotated were used to predict novel miRNA using the software Mireap (http://sourceforge.net/projects/mireap/) developed by the BGI. Additionally, the characteristic structures of miRNA precursors, including hairpins, secondary structures, Dicer cleavage sites and the minimum free energy, were used to predict novel miRNAs with the MIREAP pipeline (https://sourceforge.net/projects/mireap/). The criteria included hairpin miRNAs that can fold into necessary secondary structures and mature miRNAs that are present in one arm of the hairpin precursors. Additionally, the free energy of hybridization must be lower than or equal to −18 kcal/mol, and the mature miRNA strand and its complementary strand (miRNA*) must contain 2-nt 3' overhangs.

### Target prediction and identification

We identified targets by degradome sequencing [26]. Briefly, we matched the degraded fragments to the apple genome (*Malus × domestica* Borkh.) and removed ncRNAs, as well as polyN fragments, in the samples to reduce interference. We then used PairFinder software developed by the BGI degradome group to predict potential mRNA-miRNA pairings (Additional file 1). To predict potential functions of the putative miRNA targets in various biological processes, molecular functions and cellular components we used gene ontology (GO) categories (http://www.geneontology.org/) to classify the identified target genes [27]. Additionally, the KEGG database (fttp://fttp.genome.jp/pub/kegg/pathway/) was used for KEGG pathway analyses.

### qRT-PCR validation of miRNAs and their targets

cDNAs of miRNAs and targets were generated from 2 μg of total RNAs of 24 *M. hupehensis* samples (leaf tissue at 3, 4, 5, 6, 7 and 8 months, and the top leaves of 1-, 2-, 3-, 4-, 5- and 6-year-old trees, as well as roots, stems, flowers and fruit in June) using miRcute miRNA cDNA (Tiangen, Beijing) and PrimeScript™ RT reagent Kit with gDNA Eraser (Takara) (Figure 1). qRT-PCR was performed using a miRNA qPCR Detection Kit (SYBR Green) with 10 μl of 2X miRcute miRNA premix with ROX and SYBR green (Tiangen), and 0.4 μM of forward and reverse primers in a 20-μl system for the expression of miRNAs. PCR was also performed using SYBR® Premix Ex Taq™ II (Tli RNaseH Plus) with 10 μl of 2X SYBR® Premix Ex Taq II, and 0.8 μl of forward and reverse primers in a 20-μl system to determine the expression of the targets (Takara). The reactions were incubated in a Bio-Rad (iCycler iQ5) for 30 s at 95°C, followed by 40 cycles of 5 s at 95°C and 35 s at 60°C, followed by 81 cycles for the melt curve. Each reaction was performed in three replicates. All primers used in the qRT-PCR experiments are listed in Additional file 2.

### Leaf morphology characteristics and hormone contents

The juvenile leaves from the bases of the trees and the adult leaves from the tops of the trees were used to measure and calculate the length, width, area and dry weight (Figure 1A and D). The methods of hormone extraction and determination in leaves were carried out as previously described [28, 29].

## Results

### Construction and sequencing of small RNA and degradome libraries

**Figure 2:**
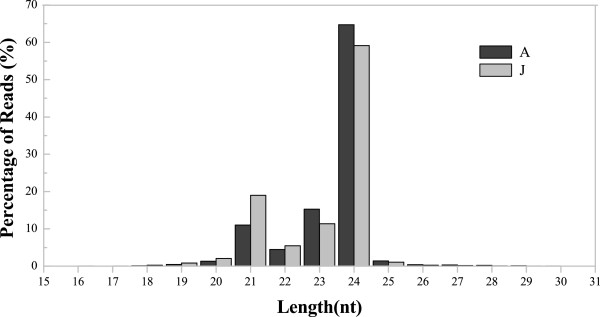
**Length distribution of small RNAs from adult and juvenile** ***Malus hupehensis*** **leaves.** The length distributions of high-quality sequences were obtained from A and J libraries. The distributions of the total reads are shown as percentages. A: Adult phase leaves from the tree top; J: Juvenile phase leaves from the tree base.

To determine responsive sRNAs in the juvenile and adult vegetative phases, A and J miRNA libraries were constructed and sequenced A total of 29,945,580 raw reads were generated by the high-throughput Illumina HiSeq. 2000 Sequencing System, with 16,316,909 and 13,628,671 reads from the A and J libraries, respectively (Table 1). After processing primary reads, 16,220,576 (99.74%) and 13,547,321 (99.68%) total clean reads were selected from the A and J libraries, respectively. We also constructed a degradome library using total RNA from the J sample (Additional file 3). The size distributions of the reads in the A and J libraries were quite similar, but there was at least 50% more 21-nt length reads in the juvenile library compared with the adult library (Figure 2). The length of the sRNA varied from 18 to 28 nt in the samples, and 21-, 23- and 24-nt small RNAs formed the major population with 24 nt being the most dominant, which is similar to the results obtained from most tested plants, including *A. thaliana* and *Brassica juncea*[30–32]. The sequencing data have been deposited in NCBI Sequence Read Archive (SRA, http://www.ncbi.nlm.nih.gov/Traces/sra_sub/sub.cgi). And accession number was SRP048848. Meanwhile, related data have been already deposited in Gene Expression Omnibus (GEO; http://www.ncbi.nlm.nih.gov/geo/query/acc.cgi?acc=GSE63373). And accession number was GSE63373.

**Table 1:** **Raw and clean read statistics of small RNAs isolated from** ***Malus hupehensis*** **leaves**

	A		J	
	Count	%		Count
total_reads	16316909		total_reads	16316909
high_quality	16262057	100%	high_quality	16262057
3′adapter_null	7741	0.05%	3′adapter_null	7741
insert_null	2554	0.02%	insert_null	2554
5′adapter_contaminants	25157	0.15%	5′adapter_contaminants	25157
smaller_than_18nt	5041	0.03%	smaller_than_18nt	5041
polyA	988	0.01%	polyA	988
clean_reads	16220576	99.74%	clean_reads	16220576

**Figure 3:**
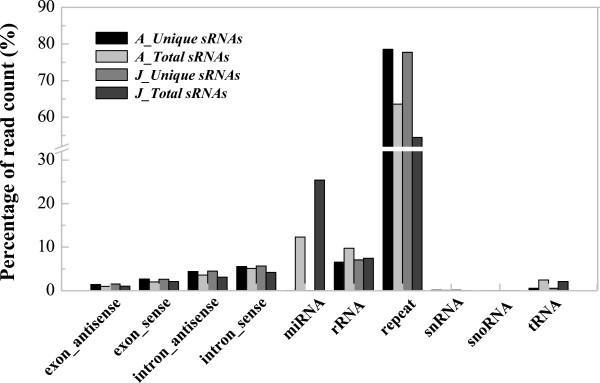
**Classification of unique small RNA reads from adult and juvenile** ***Malus hupehensis*** **leaves.** The reads were categorized into 10 different classes of small RNAs with their distributions as percentages. A: Adult phase leaves from the tree top; J: Juvenile phase leaves from the tree base.

The sRNA reads were grouped based on their identities (4,912,555 for A and 3,652,984 for J, respectively) as determined by mapping them to the domesticated apple’s genome (*Malus × domestica* Borkh.) using SO*AP2* software [33]. Approximately, a half and a third of the unique sRNA sequences for A and J matched this genome, respectively (Table 2). The reads were categorized into different classes of sRNAs, including rRNA, miRNA, snRNA, snoRNA and repeats, by matching them with the domesticated apple’s genome in the Rfam (http://www.sanger.ac.uk/resources/databases/rfam.html) and GenBank (http://www.ncbi.nlm.nih.gov/genbank) databases using the tag2annotation software. The repeats, miRNAs and rRNAs formed the major population, and repeats were the most dominant class of sRNA; however, the majority of sRNAs remained unannotated. For almost every type of sRNA, a number of unique sRNAs were shared between the A and J libraries (Figure 3).

**Table 2:** **Mapping statistics of small RNAs isolated from** ***Malus hupehensis*** **leaves**

Libraries	Unique sRNAs	Percentage(%)	Total sRNAs	Percentage(%)
A	4912555	47.83	5754465	19.33
J	3652984	35.57	4095344	13.76

### Known miRNA and expression levels

**Figure 4:**
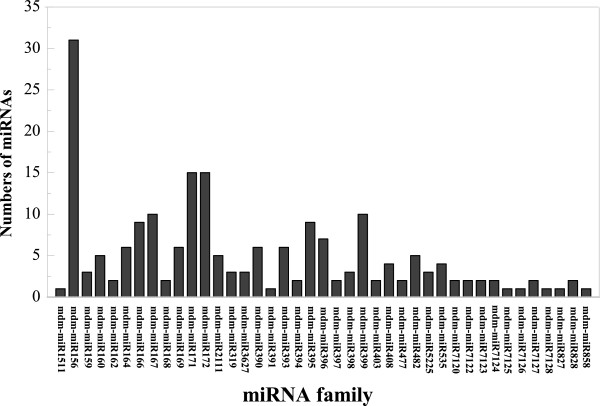
**Numbers of identified miRNAs in known miRNA families in** ***Malus hupehensis.***

To identify the known miRNAs in *M. hupehensis* (Pamp.) Rehd., the sRNAs in the two libraries were queried using BLASTN to known mature plant miRNAs of *M. domestica* in the miRBase 18.0 (http://www.mirbase.org) and plant miRNA (http://bioinformatics.cau.edu.cn/PMRD) databases [25]. A total of 207 known miRNAs belonging to 42 miRNA families were identified (Additional file 4). Different sequences are cloned into libraries at different frequencies, so the read numbers of different miRNA species may be biased by the methods of miRNAs library construction. This potential problem can be addressed by using a new method of miRNAs library construction, increasing the sample size or improving the accuracy of the data analysis and comparisons (Additional file 4). The number of members within different miRNA families varied significantly. A majority of the 42 known miRNA families had several members, and five families, mdm-miR156, mdm-miR171, mdm-miR172, mdm-miR167 and mdm-miR399, had 31, 15, 15, 10 and 10 members, respectively. Seven of the known miRNA families, mdm-miR1511, mdm-miR391, mdm-miR7125, mdm-miR7126, mdm-miR7128, mdm-miR827 and mdm-miR858, had only one member (Figure 4).

The expression levels of known miRNAs were determined by their sequence count frequencies in the A and J libraries. Some miRNAs showed library-specific expression patterns and the frequency of members from the same or different miRNA families varied greatly in each library, ranging from one to 446. Additionally, the expression levels of some miRNAs, including mdm-miR159, 164, 172, 319, 477 and 827, were found at very low levels, with read counts ranging from 0 to 10 between the A and J libraries. Others, including mdm-miR156, 166, 167, 168, 408 and 391, had high expression levels, with read counts that reached more than 10,000 in each library (Additional file 4).

**Figure 5:**
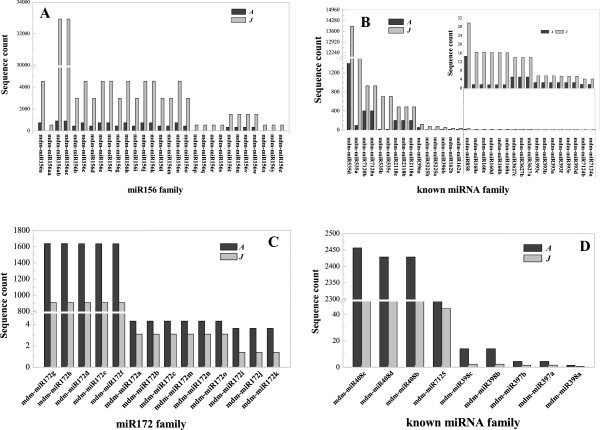
**Differentially expressed known miRNA families from libraries of adult and juvenile** ***Malus hupehensis*** **leaves.** The differentially expressed known miRNAs are shown for the A and J libraries. The expression levels are given as sequence counts. A: Adult phase leaves from the tree top; J: Juvenile phase leaves from the tree base. miR156 family numbers **(A)** and other known miRNA family numbers **(B)** expressed higher in J than in A; miR172 family numbers **(C)** and other known miRNA family numbers **(D)** expressed higher in A than in J.

Analyzing known miRNA expression levels between the A and J libraries revealed that the 17 known miRNA families (mdm-miR156, 172, 398, 397, 7125, 408, 160, 7124, 393, 3627, 5225, 396, 858, 535, 162, 2118 and 7120) were differentially expressed (Figure 5). Among these, the expression levels of mdm-miR156 and 11 other miRNA family members, mdm-miR160, 7124, 393, 3627, 5225, 162, 2118, 7120, 396, 858 and 535, in the J library were significantly higher than in the A library (Figure 5A,B). However, the expression levels of mdm-miR172 and four other miRNA family members, mdm-miR398, 397, 7125 and 408, were significantly higher in the A library than in the J library (Figure 5C,D). We also found that mdm-miR1511, 159, 164, 166, 167, 168, 171, 390, 191, 395, 403, 483, 7121, 7122, 7123 and 7126 showed no significant differences in expression levels between the A and J libraries (Additional file 4). The different expression patterns of known miRNAs in the A and J libraries may reflect a divergence in their potential biological functions during the phase transition from vegetative growth to reproductive growth.

### Putative novel miRNA in *M. hupehensis*

The reference genome sequences of the domesticated apple (*Malus × domestica* Borkh.) were used to predict potential novel miRNAs. In this study, several miRNA characteristics, including the miRNA precursor’s hairpin structure, which was predicted by the software Mireap (http://sourceforge.net/projects/mireap/), the Dicer cleavage site and the minimum free energy of the unannotated sRNA tags, which could be mapped to the genome, were used to identify putative novel miRNAs. We also used 10 reads per million as a cutoff to eliminate miRNAs with low expression levels. We identified 172 putative unique *M. hupehensis* (Pamp.) Rehd. miRNAs in the A and J sRNA libraries (Additional file 5). The lengths of the predicted novel miRNA hairpin structures ranged from 61 to 242 nt. We also found that a majority of identified novel miRNA sequences were at the 5′-ends of the hairpins rather than the 3′-ends (Additional file 5).

**Figure 6:**
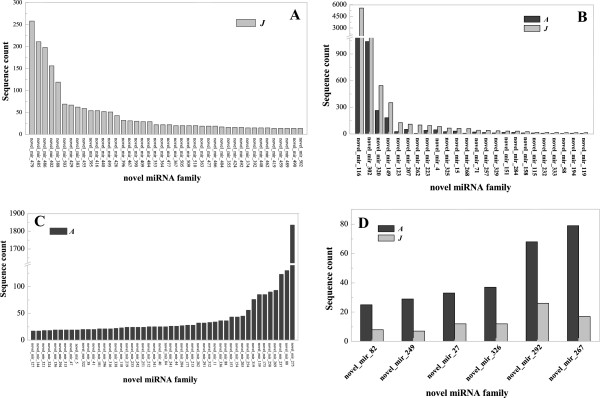
**The differentially expressed novel miRNAs from libraries of adult and juvenile** ***Malus hupehensis*** **leaves.** The differentially expressed novel miRNAs are shown for the A and J libraries. The expression levels are given as sequence counts. A: Adult phase leaves from the tree top; J: Juvenile phase leaves from the tree base. Novel miRNA family numbers expressed in J **(A)** and expressed higher in J than in A **(B)**; novel miRNA family numbers expressed in A **(C)** and expressed higher in A than in J **(D)**.

Of the 172 novel miRNAs, 31 were highly expressed in at least one library (more than 100 reads per million), and novel_miR22, novel_miR276 and novel_miR275 were the most highly expressed novel miRNAs (Additional file 6). When comparing the expression levels of these novel miRNAs between the A and J libraries, some of the putative novel miRNAs showed distinctive expression profiles. For example, 42 were expressed only in the J library, including novel_miR479, 485, and 388 (Figure 6A). The expression of another 48, including novel_miR127, 144, 204, 312, 275, 254 and 282, were detected only in the A library (Figure 6C). Additionally, 21 novel miRNAs, including novel_miR116, 302, 320, 149, 123, 207 and 262, were expressed significantly higher in the J library than in the A library (Figure 6B); however, the expression levels of novel_miR82, 249, 27, 326, 292 and 326 were significantly lower in the J library than in the A library (Figure 6D). We also found that another 52 novel miRNAs, including novel_miR343, 253, 133, 273, 128 130, 260, 17, 244 and 290, were similarly expressed in both the A and J libraries (Additional file 5). In summary, our results showed that the known and novel miRNAs presented highly diverse expression patterns between the libraries, indicating that they may play different roles in phase-associated biological processes.

### Targets of known and novel miRNAs

To explore the functions of the identified miRNAs, known and novel, in diverse biological processes and obtain insights into the mechanisms of the juvenile to adult transition in apple trees, we identified their targets by degradome sequencing (Tables 3 and 4; Additional file 1). In total, 127 targets of 25 known miRNA families, including mdm-miR156, mdm-miR159, mdm-miR166 and mdm-miR172, were detected in our library (Table 3; Additional file 6). The distributions of known miRNAs and their target cleavage sites are shown in an Additional file 6. We also detected 168 unique targets of 35 unique novel miRNAs (Table 4; Additional file 7). The distributions of novel miRNAs and their target cleavage sites are shown in an Additional file 7. In most cases, a single miRNA regulated multiple targets. For example, mdm-miR156 regulated 15 genes, including the *SBP* domain, *SPL2*, *SPL9* and acyl-CoA synthetase5. However, in some cases, single genes were targeted by several miRNAs. The *MYB* domain protein 65 was targeted by both mdm-miR159 and mdm-miR319, and the autoinhibited Ca2 + −ATPase was regulated by both mdm-miR858 and mdm-miR3627. The known miRNA targets included some *TFs*, including *SPL2* (mdm-miR156), *SPL9* (mdm-miR156), *ARF16* (mdm-miR160) and *MYB5* (mdm-miR858), and others contained several regulatory proteins, including the *LETM1-LIKE* protein (mdm-miR162), AUX signaling F-box 2 protein (mdm-miR393) and the AT hook motif DNA-binding family protein (mdm-miR3627) (Table 3). We also identified several novel miRNA targets, including some regulatory proteins and some TFs. For example, novel_miR329, novel_miR157 and novel_miR413 targeted genes encoding a C2H2-like zinc finger protein, the MYB domain protein105 and a SBP family protein, respectively. Additionally, novel_miR169 targeted the RING/U-box and integrase-type DNA-binding superfamily proteins (Table 4).

**Table 3:** **Potential targets of the identified known miRNAs from** ***Malus hupehensis*** **leaves by degradome analysis**

miRNA	Target protein	Target function	At Locus	Accession ID
mdm-miR156	Squamosa promoter-binding protein-like	transcription factor	AT1G69170.1	MDP0000119458 ;MDP0000778465;MDP0000589558
	(*SBP* domain) transcription factor family protein			MDP0000142582; MDP0000146640; MDP0000171877
				MDP0000176265 ;MDP0000193702; MDP0000210138
				MDP0000246046 ;MDP0000263766
mdm-miR156	squamosa promoter binding protein-like 2(*SPL2*)	transcription factor	AT5G43270.1	MDP0000155354
mdm-miR156	squamosa promoter binding protein-like 9(*SPL9*)	transcription factor	AT2G42200.1	MDP0000297978;MDP0000322647
mdm-miR156	acyl-CoA synthetase 5		AT1G62940.1	MDP0000249364
mdm-miR159	myb domain protein 65	transcription factor	AT3G11440.1	MDP0000147309
mdm-miR160	auxin response factor 16	transcription factor	AT4G30080.1	MDP0000131481; MDP0000221322; MDP0000750392
mdm-miR160	auxin response factor 17	transcription factor	AT1G77850.1	MDP0000232116; MDP0000256621
mdm-miR160	Co-chaperone GrpE family protein		AT1G36390.1	MDP0000273491
mdm-miR162	LETM1-LIKE protein		AT3G11560.2	MDP0000187512
mdm-miR164	NAC domain containing protein 1		AT1G56010.2	MDP0000298182; MDP0000528658
mdm-miR166	homeobox gene 8		AT4G32880.1	MDP0000005879; MDP0000126553
mdm-miR166	Homeobox-leucine zipper family protein/ lipid-		AT2G34710.1	MDP0000050082;MDP0000943529
	binding START domain-containing protein		AT5G60690.1	MDP0000236500; MDP0000242861; MDP0000426630
			AT1G52150.3	MDP0000251484
			AT1G52150.1	MDP0000313059
mdm-miR167	auxin response factor 6	transcription factor	AT1G30330.2	MDP0000319957; MDP0000550049
			AT1G30330.1	MDP0000153538
mdm-miR167	auxin response factor 8	transcription factor	AT5G37020.1	MDP0000137461; MDP0000232417
mdm-miR167	auxin response factor 19	transcription factor	AT1G19220.1	MDP0000268306
mdm-miR168	Stabilizer of iron transporter SufD/ Polynucleotidyl transferase		AT1G48410.1	MDP0000069525; MDP0000161046; MDP0000305971
mdm-miR169	nuclear factor Y, subunit A7	transcription factor	AT1G30500.2	MDP0000164531; MDP0000183865
			AT1G30500.1	MDP0000279028
mdm-miR169	nuclear factor Y, subunit A9	transcription factor	AT3G20910.1	MDP0000146933
mdm-miR169	translocation protein-related		AT5G12840.1	MDP0000296077
mdm-miR171	GRAS family transcription factor	transcription factor	AT4G00150.1	MDP0000151144 ;MDP0000275704 ;MDP0000274120
			AT2G45160.1	MDP0000784909
mdm-miR172	related to *AP2*.7	transcription factor	AT2G28550.3	MDP0000163645; MDP0000181606; MDP0000200319;
				MDP0000296716
mdm-miR172	Integrase-type DNA-binding superfamily protein	transcription factor	AT4G36920.1	MDP0000137561
mdm-miR319	myb domain protein 65	transcription factor	AT3G11440.1	MDP0000147309
mdm-miR3627	AT hook motif DNA-binding family protein		AT2G45850.1	MDP0000133746;MDP0000231744
mdm-miR3627	autoinhibited Ca2+ − ATPase, isoform 8		AT5G57110.1	MDP0000258197
mdm-miR390	exocyst subunit exo70 family protein H7		AT5G59730.1	MDP0000145463
mdm-miR391	Transmembrane proteins 14C		AT3G43520.1	MDP0000207199; MDP0000244081
mdm-miR393	F-box/RNI-like superfamily protein		AT3G62980.1	MDP0000125975; MDP0000498419
mdm-miR393	auxin signaling F-box 2		AT3G26810.1	MDP0000203334; MDP0000469943
mdm-miR393	auxin signaling F-box 3		AT1G12820.1	MDP0000268652
mdm-miR395	ATP sulfurylase 1		AT3G22890.1	MDP0000263161
mdm-miR398	DC1 domain-containing protein		AT1G60420.1	MDP0000152817 ;MDP0000308890
mdm-miR398	GroES-like zinc-binding dehydrogenase family protein		AT5G43940.1	MDP0000193167
mdm-miR5225	autoinhibited Ca2+ − ATPase, isoform 8		AT5G57110.1	MDP0000258197
mdm-miR535	Papain family cysteine protease(*RD19*)		AT4G39090.1	MDP0000189200
mdm-miR858	myb domain protein 3	transcription factor	AT1G22640.1	MDP0000184538
mdm-miR858	myb domain protein 4	transcription factor	AT4G38620.1	MDP0000031172
mdm-miR858	myb domain protein 5(*MYB5*)	transcription factor	AT3G13540.1	MDP0000133817; MDP0000143276;
				MDP0000226215;MDP0000253904
mdm-miR858	myb domain protein 7	transcription factor	AT2G16720.1	MDP0000210851
mdm-miR858	myb domain protein 12	transcription factor	AT2G47460.1	MDP0000140609 ;MDP0000887107
mdm-miR858	myb domain protein 66		AT5G14750.1	MDP0000124555
mdm-miR858	Duplicated homeodomain-like superfamily protein		AT5G35550.1	MDP0000318013; MDP0000437717
mdm-miR858	high response to osmotic stress 10		AT1G35515.1	MDP0000931057

**Table 4:** **Potential targets of the identified novel miRNAs from** ***Malus hupehensis*** **leaves by degradome analysis**

miRNA	Target protein	Target function	At Locus	Accession ID
novel_mir_106	Late embryogenesis abundant (LEA) hydroxyproline rich glycoprotein family		AT2G46150.1	MDP0000405151
novel_mir_108	RNA-binding (RRM/RBD/RNP motifs) family protein		AT2G44710.1	MDP0000242219;MDP0000303270
novel_mir_11	Flavin-containing monooxygenase family protein		AT1G48910.1	MDP0000138851;MDP0000208234
novel_mir_157	myb domain protein 105	transcription factor	AT1G69560.1	MDP0000136541; MDP0000146675
novel_mir_116	autoinhibited Ca2+ − ATPase, isoform 8		AT5G57110.1	MDP0000258197
novel_mir_156	phosphate transporter 1;4		AT2G38940.1	MDP0000141330; MDP0000523104
novel_mir_156	phosphate transporter 1;7		AT3G54700.1	MDP0000746621
novel_mir_160	Transmembrane proteins 14C		AT3G43520.1	MDP0000207199; MDP0000244081
novel_mir_169	RING/U-box superfamily protein		AT3G05200.1	MDP0000909888
novel_mir_169	Integrase-type DNA-binding superfamily protein		AT3G23230.1	MDP0000930655
novel_mir_207	phytosulfokine 4 precursor		AT3G49780.1	MDP0000145144 ;MDP0000509438; MDP0000824044
novel_mir_261	cell elongation protein / dwarf1 / diminuto (dim)		AT3G19820.1	MDP0000278275; MDP0000682675
novel_mir_262	nuclear factor Y, subunit A9	transcription factor	AT3G20910.1	MDP0000146933
novel_mir_262	nuclear factor Y, subunit A7	transcription factor	AT1G30500.2	MDP0000164531 ;MDP0000183865
			AT1G30500.1	MDP0000279028
novel_mir_262	nuclear factor Y, subunit A1	transcription factor	AT5G12840.1	MDP0000296077
novel_mir_268	nuclear factor Y, subunit A7	transcription factor	AT1G30500.2	MDP0000164531 ;MDP0000183865
			AT1G30500.1	MDP0000279028
novel_mir_27	plasmodesmata-located protein 2		AT1G04520.1	MDP0000412849
novel_mir_301	phospholipid/glycerol acyltransferase family protein		AT1G32200.1	MDP0000171689 ;MDP0000532750
novel_mir_335	SOS3-interacting protein 4		AT2G30360.1	MDP0000127732; MDP0000146449
novel_mir_351	response regulator 2		AT4G16110.1	MDP0000228719
novel_mir_37	Translation initiation factor IF6		AT3G55620.1	MDP0000247249
novel_mir_374	S-adenosyl-l-homocysteine (SAH) hydrolase 2		AT3G23810.1	MDP0000212365; MDP0000679173
novel_mir_378	ATP binding; valine-tRNA ligases; nucleotide binding; aminoacyl-tRNA ligases		AT5G16715.1	MDP0000155593; MDP0000238240
novel_mir_384	S-adenosyl-l-homocysteine (SAH) hydrolase 2		AT3G23810.1	MDP0000212365; MDP0000679173
novel_mir_392	nuclear factor Y, subunit A9	transcription factor	AT3G20910.1	MDP0000146933
novel_mir_392	nuclear factor Y, subunit A7	transcription factor	AT1G30500.2	MDP0000164531; MDP0000183865
			AT1G30500.1	MDP0000279028
novel_mir_392	nuclear factor Y, subunit A1	transcription factor	AT5G12840.1	MDP0000296077
novel_mir_422	related to *AP2* 4	transcription factor	AT1G78080.1	MDP0000401140; MDP0000633218
novel_mir_446	S-methyl-5-thioribose kinase		AT1G49820.1	MDP0000148984; MDP0000278395; MDP0000234656
novel_mir_477	aldehyde dehydrogenase 2B4		AT3G48000.1	MDP0000159395; MDP0000221713
novel_mir_477	5\′-3\′ exonuclease family protein		AT1G34380.2	MDP0000259472
novel_mir_477	OPC-8:0 CoA ligase1		AT1G20510.1	MDP0000716496
novel_mir_486	phospholipase D delta		AT4G35790.2	MDP0000125742
novel_mir_486	zinc knuckle (CCHC-type) family protein		AT5G43630.1	MDP0000147872; MDP0000196131
novel_mir_492	BRI1-associated receptor kinase		AT4G33430.1	MDP0000287771; MDP0000291093 ;MDP0000309283
novel_mir_495	ENTH/VHS/GAT family protein		AT1G06210.1	MDP0000320808
novel_mir_506	GDSL-like Lipase/Acylhydrolase superfamily protein		AT3G26430.1	MDP0000182713
novel_mir_89	NB-ARC domain-containing disease resistance protein		AT3G07040.1	MDP0000137113; MDP0000142444 ;MDP0000249156
				MDP0000662922; MDP0000242361
novel_mir_89	NB-ARC domain-containing disease resistance protein		AT3G14470.1	MDP0000206335; MDP0000241462 ;MDP0000243301
novel_mir_89	LRR and NB-ARC domains-containing disease resistance protein		AT3G14460.1	MDP0000196621
novel_mir_413	Squamosa promoter-binding protein-like (*SBP* domain) transcription factor family protein	transcription factor	AT1G69170.1	MDP0000119458; MDP0000146640; MDP0000171877
				MDP0000193702; MDP0000246046 ;MDP0000589558
				MDP0000778465
			AT5G50670.1	MDP0000142582; MDP0000176265; MDP0000210138
				MDP0000263766
novel_mir_329	myb domain protein 105	transcription factor	AT1G69560.1	MDP0000146675
novel_mir_329	C2H2-like zinc finger protein		AT1G75710.1	MDP0000179049
novel_mir_329	Frigida-like protein		AT5G27220.1	MDP0000179887
novel_mir_329	RNA-binding protein		AT2G43970.1	MDP0000266270

### GO and KEGG analyses of the degradome predicted target genes

A total of 58 GO terms from various biological processes were identified (Additional file 8). A total of 123 known miRNA targets were associated with processes such as the growth, regulation of developmental processes, hormone-mediated signaling pathways, and organ, flower and reproductive developmental processes, which are thought to be associated with the juvenile to adult transition. To better understand their biological functions, we also identified 44 GO terms for the predicted targets of the putative novel miRNAs (Additional file 9). They were associated with plant growth and development.

The GO analysis also revealed that the known miRNAs’ potential targets were associated with the biological processes of the juvenile to adult transition (Table 5). For example, the targets of mdm-miR156 and mdm-miR160 were associated with growth and flower development in plants. The targets of mdm-miR160 and mdm-miR393 were associated with AUX- and hormone-mediated signaling pathways, which play important roles in the reproductive growth of plants.

**Table 5:** **GO analyses showing that miRNAs from** ***Malus hupehensis*** **leaves potentially target the juvenile to adult transition-related biological processes**

miRNAs	GO biological process	GO ID	P value	Targets	Target number
156 ,160,162,398	growth	GO:0040007	0.0005	MDP0000146640,MDP0000155354,MDP0000131481,MDP0000221322;	8
				MDP0000256621, MDP0000187512, MDP0000308890,MDP0000193167	
166,167,393	developmental maturation	GO:0021700	2.60E-20	MDP0000005879; MDP0000126553,MDP0000319957,MDP0000137461	7
				MDP0000125975,MDP0000203334,MDP0000268652	
162,166,398	regulation of developmental growth	GO:0048638	1.20E-12	MDP0000187512,MDP0000943529,MDP0000426630,MDP0000308890	5
				MDP0000193196	
156, 162,166	developmental growth	GO:0048589	1.10E-09	MDP0000119458,MDP0000155354,MDP0000249364,MDP0000187512	5
				MDP0000005879	
160,164,167,393	auxin mediated signaling pathway	GO:0009734	1.90E-18	MDP0000131481,MDP0000221322,MDP0000256621,MDP0000298182;	8
				MDP0000528658,MDP0000319957,MDP0000125975,MDP0000203334	
159,160, 393	hormone-mediated signaling pathway	GO:0009755	5.10E-10	MDP0000147309,MDP0000131481,MDP0000221322,MDP0000125975,	6
				MDP0000203334,MDP0000268652	
159,160,399,858	response to abscisic acid stimulus	GO:0009737	3.0E-10	MDP0000147309,MDP0000131481,MDP0000256621,MDP0000143276	6
				MDP0000166425,MDP0000318013	
164,166,167,393	response to auxin stimulus	GO:0009733	3.10E-11	MDP0000298182,MDP0000005879,MDP0000319957,MDP0000125975	6
				MDP0000203334,MDP0000268652	
156,169,172,393	flower development	GO:0009908	6.30E-22	MDP0000146640,MDP0000155354,MDP0000297978,MDP0000164531,	9
				MDP0000296716,MDP0000137561,MDP0000125975,MDP0000203334	
				MDP0000268652	
160,166,167	floral organ development	GO:0048437	2.00E-23	MDP0000131481,MDP0000221322,MDP0000256621,MDP0000153538	5
				MDP0000268306	
156,393,858	androecium development	GO:0048466	1.60E-10	MDP0000146640,MDP0000155354,MDP0000297978,MDP0000249364,	14
				MDP0000125975,MDP0000203334,MDP0000268652,MDP0000143276	
				MDP0000140609.MDP0000931057	
156,393,858	stamen development	GO:0048443	1.60E-10	MDP0000146640,MDP0000155354,MDP0000297978,MDP0000249364,	14
				MDP0000125975,MDP0000203334,MDP0000268652,MDP0000143276	
				MDP0000140609.MDP0000931057	
172	specification of floral organ identity	GO:0010093	6.90E-06	MDP0000163645,MDP0000181606, MDP0000200319,MDP0000296716	5
				MDP0000137561	
156	anther development	GO:0048653	2.40E-05	MDP0000146640; MDP0000171877,MDP0000155354,MDP0000297978	6
				MDP0000322647,MDP0000249364	
172	floral organ formation	GO:0048449	0.00099	MDP0000163645,MDP0000181606, MDP0000200319,MDP0000296716	5
				MDP0000137561	
172	floral organ morphogenesis	GO:0048444	0.0038	MDP0000163645,MDP0000181606, MDP0000200319,MDP0000296716	5
				MDP0000137561	
166,167,169,858	regulation of flower development	GO:0009909	0.00012	MDP0000050082,MDP0000319957,MDP0000164531,MDP0000143276	5
				MDP0000887107	
156,160,164,166,	reproductive structure development	GO:0048608	8.20E-17	MDP0000146640,MDP0000155354,MDP0000297978,MDP0000131481,	8
				MDP0000221322,MDP0000256621,MDP0000298182,MDP0000005879	
167,168,169,172	reproductive process	GO:0022414	2.70E-13	MDP0000153538,MDP0000137461, MDP0000232417,MDP0000069525,	7
				MDP0000161046,MDP0000164531,MDP0000296716	
3627,393,398,858	reproductive developmental process	GO:0003006	3.70E-17	MDP0000133746,MDP0000258197,MDP0000125975,MDP0000203334	8
				MDP0000268652,MDP0000308890,MDP0000193167,MDP0000143276	
159,172	sexual reproduction	GO:0019953	0.14	MDP0000147309,MDP0000163645,MDP0000181606,MDP0000200319,	6
				MDP0000296716,MDP0000137561	
172	specification of organ identity	GO:0010092	6.90E-06	MDP0000163645,MDP0000181606, MDP0000200319,MDP0000296716	5
				MDP0000137561	
160,162,164	organ development	GO:0048513	3.10E-26	MDP0000131481, MDP0000221322,MDP0000256621,MDP0000273491,	7
				MDP0000187512,MDP0000298182, MDP0000528658	
156,159, 858	leaf development	GO:0048366	5.90E-12	MDP0000146640,MDP0000155354,MDP0000297978,MDP0000249364,	6
				MDP0000147309, MDP0000143276	
156, 3627,393	shoot development	GO:0048367	4.60E-13	MDP0000155354,MDP0000297978,MDP0000133746,MDP0000258197,	7
				MDP0000125975,MDP0000203334,MDP0000268652	
159,166, 858	leaf morphogenesis	GO:0009965	4.60E-13	MDP0000147309,MDP0000005879,MDP0000050082,MDP0000133817,	5
				MDP0000143276	
166,167,168	xylem and phloem pattern formation	GO:0010051	2.70E-14	MDP0000005879,MDP0000319957,MDP0000153538,MDP0000137461,	5
				MDP0000069525	
160, 393,828	root development	GO:0048364	7.60E-22	MDP0000131481, MDP0000221322,MDP0000256621,MDP0000125975	8
				MDP0000203334,MDP0000268652,MDP0000143276,MDP0000253904	
169,172,858	fruit development	GO:0010154	6.50E-06	MDP0000296077,MDP0000296716,MDP0000137561,MDP0000143276,	6
				MDP0000140609,MDP0000887107	
156,159,160	phyllome development	GO:0048827	1.80E-11	MDP0000155354,MDP0000297978,MDP0000249364,MDP0000147309	5
				MDP0000778465	
156,162, 858	tissue development	GO:0009888	5.30E-21	MDP0000146640,MDP0000155354,MDP0000297978,MDP0000187512,	5
				MDP0000143276	
164, 168,172	meristem development	GO:0048507	6.30E-27	MDP0000298182, MDP0000528658,MDP0000069525,MDP0000161046,	6
				MDP0000296716,MDP0000137561	
156,162,166	regulation of meristem development	GO:0048509	1.30E-08	MDP0000146640,MDP0000155354,MDP0000297978,MDP0000187512	6
				MDP0000005879,MDP0000126553	
164, 167,168	meristem structural organization	GO:0009933	8.40E-15	MDP0000298182,MDP0000550049,MDP0000232417,MDP0000069525,	5
				MDP0000305971	
162,166	meristem growth	GO:0035266	3.40E-10	MDP0000187512,MDP0000298182, MDP0000528658,MDP0000050082,	5
				MDP0000943529	

A total of 26 KEGG pathways were enriched for targets of known miRNAs (Additional file 10). The categories of plant hormone signal transduction and metabolic pathways contained the most targets, at 14.93%. The majority of the targets were involved in starch and sucrose metabolism, and plant hormone signal transduction, which play important roles in plant growth and development. A few of these targets were involved in the p53 signaling pathway, glycan degradation and purine metabolism, which may regulate metabolism and synthesis in plants. Additionally, we found that 13 of the 108 pathways containing targets of novel miRNAs were detected at significantly high abundance levels (*p < 0.05*) (Additional file 11). The majority of novel miRNA targets were associated with RNA polymerase or involved in plant-pathogen interactions and peptidoglycan biosynthesis.

### Identification by qRT-PCR of differentially expressed miRNAs and their targets in A and J leaves

**Figure 7:**
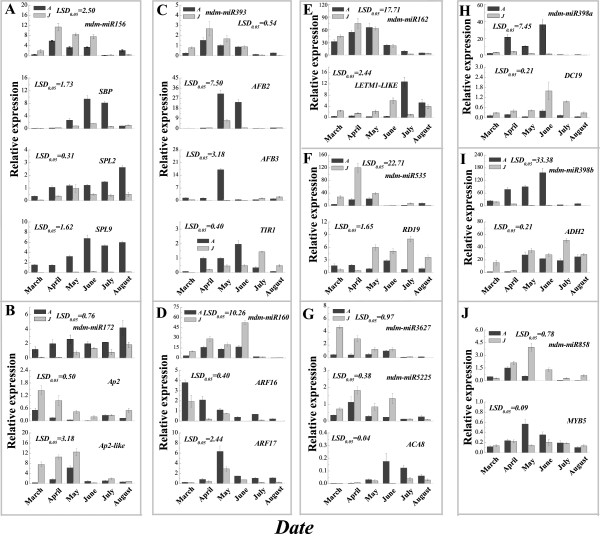
**Identification by qRT-PCR of miRNA and target expression patterns in** ***Malus hupehensis*** **leaves.** A: Adult phase leaves from the tree top; J: Juvenile phase leaves from the tree base. The expression of miRNAs and their targets in A and J leaves: mdm-miR156 **(A)**; mdm-miR172 **(B)**; mdm-miR393 **(C)**; mdm-miR160 **(D)**; mdm-miR162 **(E)**; mdm-miR535 **(F)**; mdm-miR3627and mdm-miR5225 **(G)**; mdm-miR398a **(H)**; mdm-miR398b **(I)**; and mdm-miR858 **(J)**.

To examine the expression levels of miRNAs and their targets during different developmental leaf tissue stages (3, 4, 5, 6, 7 and 8 months) between the A and J samples, as well as to confirm the sequencing results, we examined expression levels of 11 miRNAs and 16 targets by qRT-PCR (Figure 7). The up-regulation in J compared with A was confirmed for mdm-miR156 during leaf development (Figure 7A), while its targets, *SPL2* and *SPL9*, showed higher expression levels in A than in J from March to August, with their levels gradually increasing (Figure 7A). The qRT-PCR experiments also validated the deep-sequencing results of the down-regulation in J compared with A leaves for mdm-miR172, mdm-miR398a and mdm-miR398a in April, May and June (Figure 7B,H and I). The expression levels of mdm-miR172 targets *AP2* and *AP2-like* were significantly higher in J than in A leaves during early leaf development (from March to May); however, they were relatively low later (from June to August) (Figure 7B). The *DC19* (mdm-miR398a’s target) and *ADH2* (mdm-miR398b’s target) gene patterns were similar to those of *AP2* and *AP2-like* (Figure 7 H and I). mdm-miR160 and mdm-miR393 were up-regulated in J compared with A during the early leaf development stage (from March to June), while most of their targets showed significantly higher expression levels in A than in J. Additionally, we found that *AFB2* and *AFB3* (mdm-miR393’s targets) were detected in A but were almost undetectable in J leaves (May and June) (Figure 7C). The expression of *TIR1* (mdm-miR393’s target) was higher in A than in J during April, May and August, but was lower in A than in J during July and August (Figure 7C).

Hierarchical clustering of known miRNAs and targets by expression levels in the A and J leaves of *M. hupehensis* resulted in six and five major clusters, respectively (Additional file 12). The miRNAs within cluster 1 (miR172 for A and J) typically displayed high expression levels during the later stages of leaf development (August), but miRNAs within cluster 6 (miR156, 169, 393 and 858 for A and miR156 and 5225 for J) displayed opposite results, with high expression levels during the early stages (April and May) (Figure 7 and Additional file 12A). Additionally, the targets of cluster 1 (*AP2* and *ARF16* for A and J, respectively) displayed high expression levels in the early stages of leaf development (March to April); however, the cluster 5 targets (such as *SPL9*, *SBP* and *TIR1*) showed the opposite results, with higher expression levels in later stages (July and August) (Figure 7 and Additional file 12B). These data suggest that the expression patterns of miRNAs and their targets, such as miR156 and *SPL9*, display opposite trends during leaf development.

### Identification by qRT-PCR of miRNA and target expression patterns in leaves of different ages

**Figure 8:**
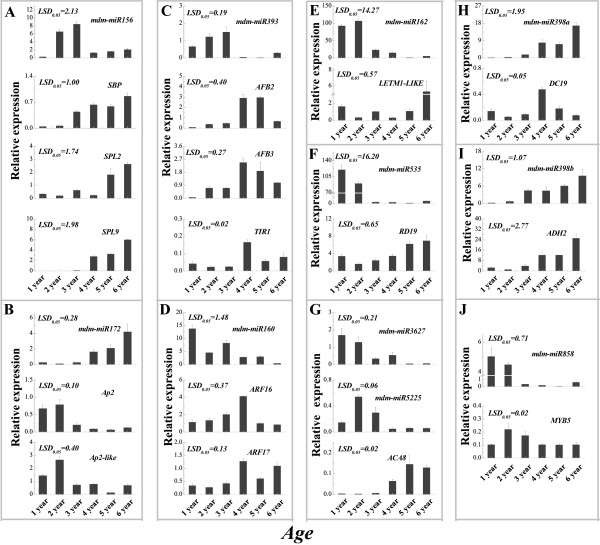
**Identification by qRT-PCR of miRNA and target expression patterns in** ***Malus hupehensis*** **leaves of different ages.** A: Adult phase leaves from the tree top; J: Juvenile phase leaves from the tree base. The expression of miRNAs and their targets in leaves of different ages: mdm-miR156 **(A)**; mdm-miR172 **(B)**; mdm-miR393 **(C)**; mdm-miR160 **(D)**; mdm-miR162 **(E)**; mdm-miR535 **(F)**; mdm-miR3627 and mdm-miR5225 **(G)**; mdm-miR398a **(H)**; mdm-miR398b **(I)**; and mdm-miR858 **(J)**.

We validated miRNA and target expression profiles in leaves from the tops of trees of different ages (1, 2, 3, 4, 5 and 6 years old) via qRT-PCR (Figure 8 and Additional file 15). mdm-miR156 had significantly higher expression levels in younger (except 1-year-olds) than in older tree leaves (4, 5, and 6 years old) (Figure 8A), whereas its targets (*SBP*, *SPL2* and *SPL9*) had relatively higher expression levels in older tree leaves (5 and 6 years old) than in younger tree leaves (1 and 2 years old) (Figure 8B). Compared with the expression profile of mdm-miRNA156, mdm-miR172 showed a high expression level in older tree leaves (4-, 5- and 6-years-old) and this expression increased gradually in 1- to 6-year-olds (Figure 8A,B). A perfect inverse expression pattern was found for its targets, *AP2* and *AP2-like* genes, which were expressed higher in younger than in older tree leaves (Figure 8B). Mdm-miR160 exhibited strong expression in 1-year-old leaves and the expression decreased gradually in 1- to 6-year-olds, whereas its targets’ (*ARF16* and *ARF17*) expression increased to their highest levels in 4-year-olds but then decreased to relatively low levels in 5- and 6-year-olds (Figure 8D). The expression of mdm-miR393 could barely be detected in older tree leaves (4-, 5- and 6-year-olds) but was relatively high in young tree leaves (1, 2 and 3 years old) (Figure 8C). Interestingly, its targets (*AFB2*, *AFB3* and *TIR1*) had an inverse expression pattern that was higher in older leaves (4 and 5 years old) (Figure 8C).

Additionally, the hierarchical cluster analysis resulted in these known miRNAs and target genes forming three major clusters each, and it also showed that the expression levels of most known miRNAs were inversely correlated with those of the corresponding targets among leaves of different ages (Additional file 13).

### Identification by qRT-PCR of miRNA and target expression patterns in different tissues

**Figure 9:**
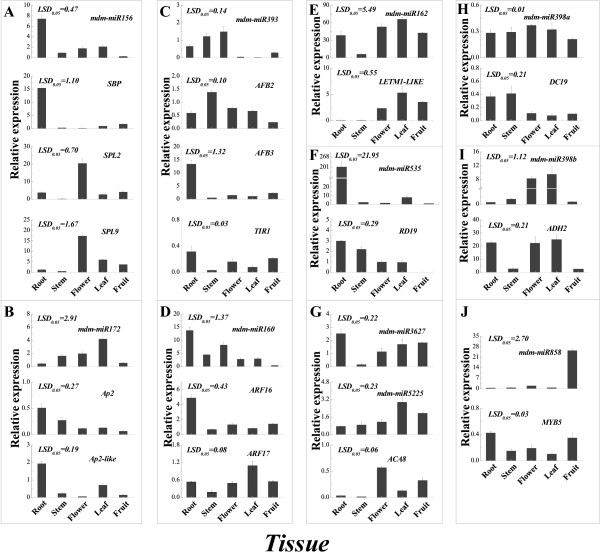
**Identification by qRT-PCR of miRNA and target expression patterns in different** ***Malus hupehensis*** **’ tissues.** A: Adult phase leaves from the tree top; J: Juvenile phase leaves from the tree base. Expression of miRNAs and their targets in different tissue: mdm-miR156 **(A)**; mdm-miR172 **(B)**; mdm-miR393 **(C)**; mdm-miR160 **(D)**; mdm-miR162 **(E)**; mdm-miR535 **(F)**; mdm-miR3627 and mdm-miR5225 **(G)**; mdm-miR398a **(H)**; mdm-miR398b **(I)**; and mdm-miR858 **(J)**.

Tissue-specific expression and hierarchical cluster analyses, which allowed known miRNAs and their target genes into three major clusters each (Additional file 14),revealed that miRNAs and their targets in different tissues (roots, stem, flower, leaves and fruit) presented a variety of expression patterns (Figure 9). The expression patterns of these miRNAs and their targets could be divided into four types: (1) mdm-miR156, mdm-miR160, mdm-miR535 and their targets, the *SBP*, *AP2*, *AP2-like*, *ARF16*, *AFB*, *DC19* and *RD19* genes, had the highest expression levels in roots but relatively low expression levels in fruit (Figure 9A,B,D,F and H); (2) mdm-miR393, mdm-miR398a, mdm-miR398b and their targets, the *SPL2*, *SPL9* and *ACA8* genes, were found to be expressed most abundantly in flowers but had relatively low expression levels in fruit and roots (Figure 9A,C,E,F,G and H); (3) mdm-miR172, mdm-miR162, mdm-miR162, mdm-miR5225 and their targets, the *ARF17*, *LETM1-LIKE* and *ADH2* genes, showed high expression levels in leaf tissue but relatively low levels were observed in stems (Figure 9B,D,E,G and I); (4) mdm-miR858 and mdm-miR3627 and their targets, the *TIR1* and *MYB5* genes, had high expression levels in fruit but low levels in stems (Figure 9C,G and J).

### Identification of novel miRNA expression patterns in *M. hupehensis*by qRT-PCR

We have verified the expression of some novel miRNAs by qRT-PCR in A and J leaves of *M. hupehensis*, as well as leaves of different ages and different tissues (Additional file 15). Novel-miR486 and novel-miR492 were detected as up-regulated in J compared with A from April to June, while novel-miR207 and novel-miR329 showed significantly higher expression levels in J than in A from May to June. We also found that novel-miR207, 329, 486 and 492 had relatively higher expression levels in leaves from young trees than from older trees (Additional files 15 and 16). A tissue-specific expression analysis revealed that novel-miR329 and 492 expressed at a higher level in flower, while novel-miR486 had the highest expression in root (Additional files 15 and 16).

### Leaf morphology characteristics and hormone content analysis

**Figure 10:**
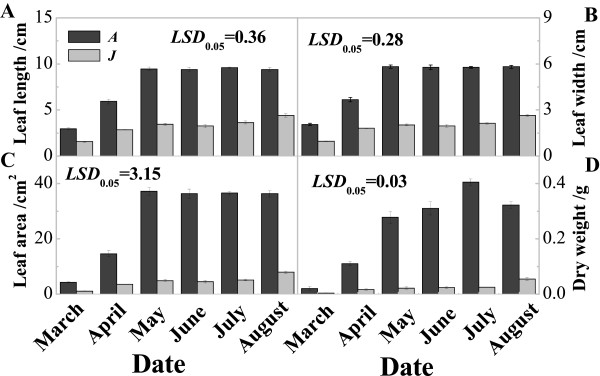
***Malus hupehensis*** **leaf characteristics in the adult and juvenile phases.** A: Adult phase leaves from the tree top; J: Juvenile phase leaves from the tree base. Leaf length **(A)**; Leaf width **(B)**; Leaf area **(C)**; and Dry weight **(D)**.

**Figure 11:**
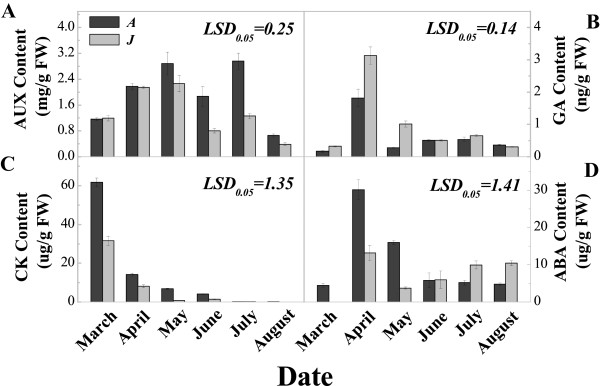
**Auxin (AUX), cytokinin (CK), gibberellic acid (GA) and abscisic acid (ABA) contents in adult and juvenile** ***Malus hupehensis*** **leaves.** A: Adult phase leaves from the tree top; J: Juvenile phase leaves from the tree base. AUX content **(A)**; GA content **(B)**; CK content **(C)**; and ABA content **(D)**.

To determine the characteristics of A and J during leaf development, we measured leaf length, width and area, and dry weight from March to August, all of which were significantly higher in A compared with J (Figure 10). Additionally, the leaf AUX content increased from March to July and then decreased in August for A. The pattern of AUX expression for J had a similar trend to A, but reached a peak in May, and the AUX content was significantly higher in A than in J during later leaf growth stages (May to August) (Figure 11A). The leaf CK content decreased gradually from March to August for both A and J, and was significantly higher in A than in J from March to June. However, the CK content was almost undetectable during July and August in both A and J leaves (Figure 11C). The leaf GA content was significantly higher in J than in A from April to May, but there was no significant difference at any other time (Figure 11B). The leaf ABA content was significantly higher in A than in J from March to May, but this was reversed in July and August (Figure 11D). The different levels of hormones between A and J leaves indicated that hormones play a vital role in leaf growth and development.

## Discussion

Leaf morphology is very different between the juvenile phase at the base of the trees and the adult phase at the top of the trees. Our results showed that leaf traits, such as size and dry weight, were significantly higher in A than in J leaves (Figure 10), which was consistent with a previous study in *A. thaliana*[7]. Plant growth hormones, such as AUXs, CKs, GA and ABA, have important roles in leaf development during the transition from vegetative to reproductive growth [34]. Spraying exogenous GAs can cause a reversion from the adult to the juvenile vegetative phase, producing juvenile traits in newly formed leaves [29, 35]. The level of GA was higher in juvenile tissue than in adult tissue of trees [35], which was consistent with our results (Figure 11). In this study, AUX, CK and ABA contents were significantly higher in A than in J leaves, but the GA content showed the opposite result, indicating that hormone levels play an important role in the physiological processes surrounding the phase transition (Figure 11).

In this study, by constructing two sRNA libraries of *M. hupehensis* (*Pamp.*) Rehd. using high-throughput sequencing, we identified 207 known miRNAs that belonged to 42 miRNA families (Additional file 4). The majority were conserved in *A. thaliana* and peach [30, 32, 36]. Among them, 12 of 17 known miRNA families had higher expression levels in the J library than in the A library (Figure 5A and B), while others showed an opposite expression pattern (Figure 5C and D). Additionally, 172 novel miRNAs were identified based on the universal rules for novel miRNA prediction and annotation [37]. Of these, 42 were only expressed in the J library (Figure 6A) and 48 were expressed only in the A library (Figure 6C). The differential expression of known and novel miRNAs may involve various biological functions in the regulation of leaf development, phase transition and reproductive growth. Using degradome analysis, we also identified a total of 127 targets for 25 known miRNA families and 168 targets for 35 unique novel miRNAs (Additional files 6 and 7). The majority of these targets were relatively conserved in other species [1] and regulated a variety of biological processes involved in developmental growth, hormone-mediated signaling pathways, flower development and reproductive processes in plants.

Because we established only one miRNA library from juvenile leaves and one from adult leaf, our results are limited and may not include all of the differences present (Figures 3, 4, 5 and 6). A study on miRNA control of vegetative phase change in the gymnosperm *Sequoia sempervirens* and the identification of sRNAs also provide evidence supporting our results [1, 18].

The GO analyses revealed that miRNA targets were mainly associated with the juvenile to adult transition during plant development (Table 5). The majority of GO terms were associated with plant growth and development (GO:0040007, GO:0021700, GO:0048638 and GO:0048589), plant tissue growth and formation (GO:0010092, GO:0048366, GO:0048367, GO:0009965 and GO:0010051), meristem development (GO:0009888, GO:0048507 and GO:0009933) and hormone-mediated signaling pathways (GO:0009734, GO:0009755, GO:0009737 and GO:0009733), which are all involved in the juvenile to adult transition-associated biological processes (Table 5). The KEGG analysis also revealed that the genes targeted by known and novel miRNAs were largely involved in starch and sucrose metabolism, plant hormone signal transduction, p53 signaling and the glycan degradation pathway, which play important roles in the juvenile to adult transition and reproductive growth in plants (Additional files 10 and 11).

It was reported that miR156 regulated leaf development, showed juvenile characteristics when overexpressed in plants and had reduced expression levels in adult leaves [12, 38]. This was consistent with our results that the expression level of mdm-miR156 in the J library was significantly higher than in the A library (Figure 5). Additionally, miR156 regulated its targets, the SPL family, through translational inhibition and gene silencing in *A. thaliana*[39]. The overexpression of the targets *SPL3*, *SPL9* and *SPL15* resulted in increased cell numbers in leaves [7, 40]. This lead to an increased leaf size in plants overexpressing *SPL3* and *SPL9*. Additionally, the overexpression of *SPL9* and *SPL15* controlled shoot maturation and leaf initiation [41]. In this study, we confirmed the up-regulation of mdm-miR156 in J leaves compared with A leaves during leaf development (Figure 7A). However, two of its targets, *SPL2* and *SPL9*, showed higher expression levels in A than in J leaves (Figures 7A and 8A). Previous research also showed that *SPL9* was expressed in the vegetative shoot apices, although the expression level of miRNA156 was almost undetectable [42]. *SPL9*, as well as *SPL10*, expression levels in the leaf primordia affect the initiation of new leaves at the shoot apical meristem [40], while expression levels of *SPL2*, along with *SPL11*, control the leaf lamina shape during shoot maturation in the reproductive phase [8]. Additionally, mdm-miR156 was highly expressed in roots but had almost no expression in flowers; however, its targets, *SPL2* and *SPL9*, were more highly expressed in flowers than in other tissues (Figure 9A). Expression levels of miRNA156 and their targets were associated with the transition from vegetative to reproductive growth and the transition to flowering.

The increased expression of miRNA156 and decreased expression of its targets (*SPLs*) delayed flowering, whereas inhibiting miR156 expression accelerated flowering [43, 44]. Some genes involved in flowering were regulated by the expression levels of miRNA156 and their targets in plants. For example, miR156 regulates *FLOWERING LOCUS T* expression in apical meristem to control temperature-responsive flowering in *A. thaliana*[45]. *LFY*, *FUL* and *AP1* genes were directly activated by the miRNA-targeted *SPL3* to control the timing of flower formation in *A. thaliana*[13]. *SPL9* and *SPL3* also directly regulated and controlled MADS-box gene expression levels that promote flowering [40]. Our results showed that leaves in the juvenile phase were much smaller and had higher miRNA156 expression levels and lower *SBP, SPL2* and *SPL9* expression levels compared with adult leaves, implying that miRNA156 and its targets may play important roles in the juvenile to adult transition, leaf development and the transition to flowering.

Previous research showed that miR172 could promote flowering, but that its targets were floral repressors, such as *AP2-like*, *AP2*, *EAT1* and *EAT2*, which play important roles in the regulation of leaf traits in *A. thaliana*[44, 46]. miR172 may be involved in regulating the juvenile to adult transition during developmental stages [47, 48]. We also found that the sit-miR172 family members’ expression levels were significantly higher in adult leaves and flower tissues in olives (*Olea europaea* L.) [47]. In our study, the expression of mdm-miR172 family members was significantly higher in the A leaf library than in the J leaf library, implying that they were active in adult stage maintenance (Figure 5C). The qRT-PCR experiments also validated the deep-sequencing results of a down-regulation of mdm-miR172 in the J library compared with the A library during leaf development (Figure 7B). However, the expression levels of the targets *AP2* and *AP2-like* (mdm-miR172 targets) were significantly higher in the J than in the A leaves from March to May (Figure 7B). These expression trends were also detected in the leaves of trees of different ages (Figure 8B). Additionally, *AP2* and *AP2-like* genes exhibited their highest expression levels in roots but had relatively low expression levels in flowers (Figure 9B). Our research showed that miRNA172 and its targets participated in the regulation of the juvenile to adult phase transition and the formation of floral organs.

Plant hormones, as major regulators, have roles in leaf growth, juvenile to adult phase transitioning and flowering [19, 49, 50]. AUX is a key hormone that is important in hormone-mediated responses during plant development [51–54]. It has been reported that AUX response factors, *ARF16* and *ARF17*, are targets of miRNA160 and regulate various biological processes of plant development in *Arabidopsis*, maize and rice [55–57]. The expression level of miRNA160 was different between the on- and off-year, and its expression was higher in juvenile leaves than in mature leaves in olive trees [47]. In our study, we also found that mdm-miR160 was up-regulated in J leaves whereas its targets were up-regulated in A leaves, supporting the high-throughput data on miRNA160 (Figures 5B, 7D). mdm-miR160 exhibited high expression levels in young leaves, but *ARF16* and *ARF17* expressed higher in the relatively mature leaves (Figure 9D). Additionally, the AUX signal F-box genes *TIR1, AFB2* and *AFB3* were negatively regulated by miRNA393 [58, 59], confirming the high-throughput sequencing and qRT-PCR results. These showed that miRNA393 expressed highly in J leaves, while its targets showed almost no expression (Figures 5B, 7C). An exogenous AUX treatment could enhance miRNA393 transcription and induce miR393 accumulation, indicating miRNA393 regulated *TIR1* through a feedback control during the plant development process [60]. Our results showed that the AUX content and the expression of the targets (*ARF16, ARF17, TIR1, AFB2* and *AFB3*) of miRNA160 and miRNA393 were significantly higher in A leaves than in J leaves (Figure 7C and D), indicating an important contribution of hormone-mediated responses to leaf maturation, reproductive growth and the flowering transition.

We also found that other miRNA family members identified by high-throughput sequencing were differentially expressed between the A and J libraries (Figure 7). These results were corroborated by qRT-PCR (Figures 7, 8 and 9). The expression profiles of mdm-miR162, mdm-miR535, mdm-miR858, mdm-miR3727 and miR5225 were up-regulated in J leaves compared with A leaves during leaf development, while mdm-miR398a and mdm-miR398b were down-regulated in J leaves compared with A leaves, implying that these miRNAs may participate in the regulating leaf development and other biological processes (Figure 7F,G,H and I). Meanwhile, the expression profiles of their targets presented opposite results for A and J leaves, which was also supported by qRT-PCR results (Figure 7F,G,H and I). This was consistent with a previous study on cotton, which showed that the expression of miRNAs (398a and miR398b) and their targets (*RD19* and *ADH2*, respectively) had opposite expression patterns [61, 62]. The expression patterns of miR858 and their targets (MYB family) were also similar to those found in apple [22] and peach trees (Figures 8 and 9) [36].

## Conclusions

A comprehensive study on *M. hupehensis* miRNAs related to the juvenile to adult phase transition was performed. In this study, we identified 42 known miRNA families and 172 novel miRNAs from two sRNA libraries. Additionally, using a degradome analysis, we identified 127 targets of the 25 known miRNA families and 168 targets of the 35 unique novel miRNAs. A GO analysis showed that these miRNAs and their targets participated in regulating phase transition and reproductive growth during plant development. Our results showed that the juvenile to adult phase transition and flowering were controlled by mdm-miRNA156 and mdm-miRNA172. mdm-miR156 is highly abundant in J leaves and decreases in A leaves, while mdm-miR172 has the opposite expression pattern in the two leave types. The miRNA-mediated regulation of multiple plant hormone pathways, such as the GA, AUX, CK and ABA, also plays key roles in phase transition and flowering during the plant life cycle. The identification of the mdm-miR160–target (*ARF16* and *ARF17*) and mdm-miR393–target (*AFB2*, *AFB3* and *TIR1*) hormone-mediated expression patterns significantly improves our understanding of the roles miRNAs play in the regulation of plant growth, development, reproductive phase transition and flowering. In general, the combination of sRNA and degradome sequencing can better illustrate the profiles of hormone-regulated miRNAs and miRNA targets involved in complex regulatory networks, thus contributing to the understanding of miRNA functions during growth, phase transition and reproductive growth in perennial woody fruit trees.

## Electronic supplementary material

Additional file 1: **Targets of known miRNAs in** ***Malus hupehensis*** **identified by degradome analysis.** (DOCX 21 KB)

Additional file 2: **Primer sequences of selected known and novel miRNAs and target genes for qRT-PCR validation experiments in** ***Malus hupehensis*** **.** (XLSX 12 KB)

Additional file 3: **The length distribution of small RNAs in the** ***Malus hupehensis*** **juvenile leaf degradome library.** (DOCX 46 KB)

Additional file 4: **Identified known miRNAs in** ***Malus hupehensis*** **and their read counts.** (DOCX 42 KB)

Additional file 5: **Identified novel miRNAs in** ***Malus hupehensis*** **and their read counts.** (DOCX 46 KB)

Additional file 6: **The total number of distinct transcripts targeted by unique known miRNAs detected in the** ***Malus hupehensis*** **.** (XLSX 55 KB)

Additional file 7: **The total number of distinct transcripts targeted by unique novel miRNAs detected in the** ***Malus hupehensis*** **.** (XLSX 34 KB)

Additional file 8: **GO functional analysis of identified targets of known miRNAs identified in the** ***Malus hupehensis*** **.** (XLSX 21 KB)

Additional file 9: **GO functional analysis of identified targets of novel miRNAs identified in the** ***Malus hupehensis*** **.** (XLSX 16 KB)

Additional file 10: **KEGG functional analysis of identified targets of known miRNAs identified in the** ***Malus hupehensis*** **.** (XLSX 12 KB)

Additional file 11: **KEGG functional analysis of identified targets of novel miRNAs identified in the** ***Malus hupehensis*** **.** (XLSX 19 KB)

Additional file 12: **Hierarchical clustering of known miRNAs (A) and targets (B) by expression levels in adult and juvenile leaves of** ***Malus hupehensis*** **.** Samples are reported on the top side of the heat map with the following codes: Date (from March to August). A: Adult phase leaves from the tree top; J: Juvenile phase leaves from the tree base. (DOCX 131 KB)

Additional file 13: **Hierarchical clustering of known miRNAs (A) and targets (B) by expression levels in** ***Malus hupehensis*** **leaves of different ages.** Samples are reported on the top side of the heat map with the following codes: Age (from 1 to 6 years). A: Adult phase leaves from the tree top; J: Juvenile phase leaves from the tree base. (DOCX 75 KB)

Additional file 14: **Hierarchical clustering of known miRNAs (A) and targets (B) by expression levels in different** ***Malus hupehensis*** **’ tissues.** Samples are reported on the top side of the heat map with the following codes: Tissues (root, stem, flower, leaf and fruit). A: Adult phase leaves from the tree top; J: Juvenile phase leaves from the tree base. (DOCX 78 KB)

Additional file 15: **Hierarchical clustering of novel miRNAs with expression levels in adult and juvenile leaves of** ***Malus hupehensis*** **(A) in leaves of different ages (B) and in different tissues (C).** Samples are reported on the top side of the heat map with the following codes: Date (From March to August) (A); Age (from 1 to 6 years) (B); Tissues (root, stem, flower, leaf and fruit) (c). A: Adult phase leaves from the tree top; J: Juvenile phase leaves from the tree base. (DOCX 83 KB)

Additional file 16: **Identification by qRT-PCR of novel miRNA expression patterns in adult and juvenile leaves of** ***Malus hupehensis*** **(A) in leaves of different ages (B) and in different tissues (C).** A: Adult phase leaves from the tree top; J: Juvenile phase leaves from the tree base. (DOCX 75 KB)

## Abbreviations

AUX: Auxin
CK: Cytokinin
ABA: Abscisic acid
GA: Gibberellic acid
A: Adult
J: Juvenile.
