# Supplementary material for: Genome-wide identification of vegetative phase transition-associated microRNAs and target predictions using degradome sequencing in Malus hupehensis
Source: BMC Genomics. 2014 Dec 17;15(1):1125. doi: 10.1186/1471-2164-15-1125 (PMC4523022; doi:10.1186/1471-2164-15-1125)
Supplement: Supplementary file 1 — Additional file 1: Targets of known miRNAs in Malus hupehensis identified by degradome analysis. (DOCX 21 KB) [file 12864_2014_7075_MOESM1_ESM.docx]

**Additional file 1. Targets of known miRNAs in Malus hupehensis identified by degradome analysis.**

| **miRNA** | **Target protein** | **Target function** | **At Locus** | **Accession ID** |
| --- | --- | --- | --- | --- |
| mdm-miR1511 | DNA topoisomerase 1 beta |  | AT5G55310.1 | MDP0000559762 |
| mdm-miR2111 | Galactose oxidase/kelch repeat superfamily protein |  | AT3G27150.1 | MDP0000417953;MDP0000399538; MDP0000892979;  MDP0000895750 |
| mdm-miR2118 | heat shock transcription factor A6B | transcription factor | AT3G22830.1 | MDP0000119199 |
| mdm-miR394 | FGGY family of carbohydrate kinase |  | AT4G30310.2 | MDP0000268873 |
| mdm-miR394 | Heavy metal transport/detoxification superfamily protein |  | AT1G71050.1 | MDP0000316006 |
| mdm-miR394 | S-adenosyl-L-methionine-dependent protein  methyltransferases superfamily |  | AT2G45750.1 | MDP0000278349 |
| mdm-miR396 | S-adenosyl-L-methionine-dependent  methyltransferases superfamily protein |  | AT3G01660.1 | MDP0000193012; MDP0000501816 |
| mdm-miR396 | NADH-dependent glutamate synthase 1 |  | AT5G53460.1 | MDP0000442206 |
| mdm-miR396 | 2-oxoglutarate (2OG) and Fe(II)-dependent  oxygenase superfamily protein |  | AT3G21360.1 | MDP0000314475; MDP0000603111 |
| mdm-miR396 | Tetratricopeptide repeat (TPR)-like superfamily protein |  | AT3G16760.1 | MDP0000297552 |
| mdm-miR396 | DNA binding;ATP binding |  | AT3G48770.1 | MDP0000677637 |
| mdm-miR397 | laccase 11 | lignin catabolic process | AT5G03260.1 | MDP0000240772 |
| mdm-miR397 | laccase 17 | lignin catabolic process | AT5G60020.1 | MDP0000175212;MDP0000244021; MDP0000237964 |
| mdm-miR397 | Glycosyl hydrolase family 38 protein |  | AT5G66150.1 | MDP0000267909 |
| mdm-miR397 | Tetratricopeptide repeat (TPR)-like superfamily protein |  | AT5G66150.1 | MDP0000250936 MDP0000891893 |
| mdm-miR397 | Caleosin-related family protein |  | AT3G09220.1 | MDP0000126274 |
| mdm-miR397 | laccase 1 | lignin catabolic process | AT1G70670.1 | MDP0000243556 |
| mdm-miR397 | laccase 7 | lignin catabolic process | AT1G18140.1 | MDP0000280305 |
| mdm-miR397 | laccase 17 | lignin catabolic process | AT3G09220.1 | MDP0000149146 |
| mdm-miR397 | Glycosyl hydrolase family 38 protein |  | AT5G60020.1 | MDP0000237964 |
| mdm-miR399 | phosphate transporter 1;1 |  | AT5G43350.1 | MDP0000166425 |
| mdm-miR399 | phosphate transporter 1;4 |  | AT2G38940.1 | MDP0000141330 |
| mdm-miR403 | UDP-Glycosyltransferase superfamily protein |  | AT1G22400.1 | MDP0000175678 |
| mdm-miR403 | MuDR family transposase |  | AT1G64260.1 | MDP0000272603 |
| mdm-miR477 | Major facilitator superfamily protein |  | AT2G39210.1 | MDP0000522880; MDP0000758845 |
| mdm-miR477 | Abscisic acid-responsive(TB2/DP1, HVA22)  family protein |  | AT5G42560.1 | MDP0000232264 |
| mdm-miR477 | vacuolar ATP synthase subunit A |  | AT1G78900.1 | MDP0000248012; MDP0000844729 |
| mdm-miR477 | recA DNA recombination family protein |  | AT2G19490.1 | MDP0000272485 |
| mdm-miR482 | LRR and NB-ARC domains-containing  disease resistance protein |  | AT3G14460.1 | MDP0000289031 MDP0000217885 |
| mdm-miR482 | NB-ARC domain-containing disease |  | AT3G14470.1 | MDP0000692851 ;MDP0000232833 ;MDP0000158225 |
|  | resistance protein |  |  | MDP0000552270 MDP0000472974 |
| mdm-miR482 | disease resistance protein(TIR-NBS-LRR class), |  | AT5G17680.1 | MDP0000258186; MDP0000641933; MDP0000136726 |
|  | putative |  |  | MDP0000136726; MDP0000378930 ;MDP0000129088 |
|  |  |  |  | MDP0000291677; MDP0000210772 |
| mdm-miR482 | Target of Myb protein 1 |  | AT1G76970.1 | MDP0000279752 |
| mdm-miR482 | RING/U-box superfamily protein |  | AT3G60080.1 | MDP0000680869; MDP0000238122 |
| mdm-miR482 | Target of AVRB operation1 |  | AT5G44510.1 | MDP0000177288 |
| mdm-miR7121 | H(+)-ATPase 2 |  | AT4G30190.1 | MDP0000150049; MDP0000259837 |
| mdm-miR7124 | Drought sensitive 1 |  | AT1G80710.1 | MDP0000239478;MDP0000162030 ;MDP0000309729 |
| mdm-miR7124 | general control non-repressible 5 |  | AT5G64840.1 | MDP0000245747 |
| mdm-miR7125 | myb domain protein 66 | transcription factor | AT3G12750.1 | MDP0000273257 |
| mdm-miR7126 | F-box/RNI-like superfamily protein |  | AT3G58900.1 | MDP0000150093 |
| mdm-miR7126 | Phototropic-responsive NPH3 family protein |  | AT5G64330.2 | MDP0000198439 |
| mdm-miR7126 | splicing factor-related |  | AT5G06160.1 | MDP0000932888 |
| mdm-miR7127 | disease resistance family protein / LRR family protein |  | AT2G34930.1 | MDP0000235802 ;MDP0000149947 |
| mdm-miR7127 | 5-formyltetrahydrofolate cycloligase |  | AT5G13050.1 | MDP0000158091 |
| mdm-miR828 | myb domain protein 5(MYB5) | transcription factor | AT3G13540.1 | MDP0000143276 ;MDP0000133817 ; |
|  |  |  |  | MDP0000253904;MDP0000226215 |
| mdm-miR828 | myb domain protein 66 | transcription factor | AT5G14750.1 | MDP0000124555 ;MDP0000317209; MDP0000193637 |
|  |  |  | AT5G14750.1 | MDP0000578193 ;MDP0000650225 ;MDP0000642761 |
|  |  |  |  | MDP0000164048 |
| mdm-miR828 | high response to osmotic stress 10 |  | AT1G35515.1 | MDP0000931057 |
| mdm-miR828 | protein phosphatase 2A subunit A2 |  | AT3G25800.1 | MDP0000145557 |
| mdm-miR828 | Duplicated homeodomain-like superfamily protein |  | AT5G35550.1 | MDP0000475808 |
