# Supplementary material for: Genome-wide identification of vegetative phase transition-associated microRNAs and target predictions using degradome sequencing in Malus hupehensis
Source: BMC Genomics. 2014 Dec 17;15(1):1125. doi: 10.1186/1471-2164-15-1125 (PMC4523022; doi:10.1186/1471-2164-15-1125)

Additional file 3. The length distribution of small RNAs in the Malus hupehensis juvenile leaf degradome library.


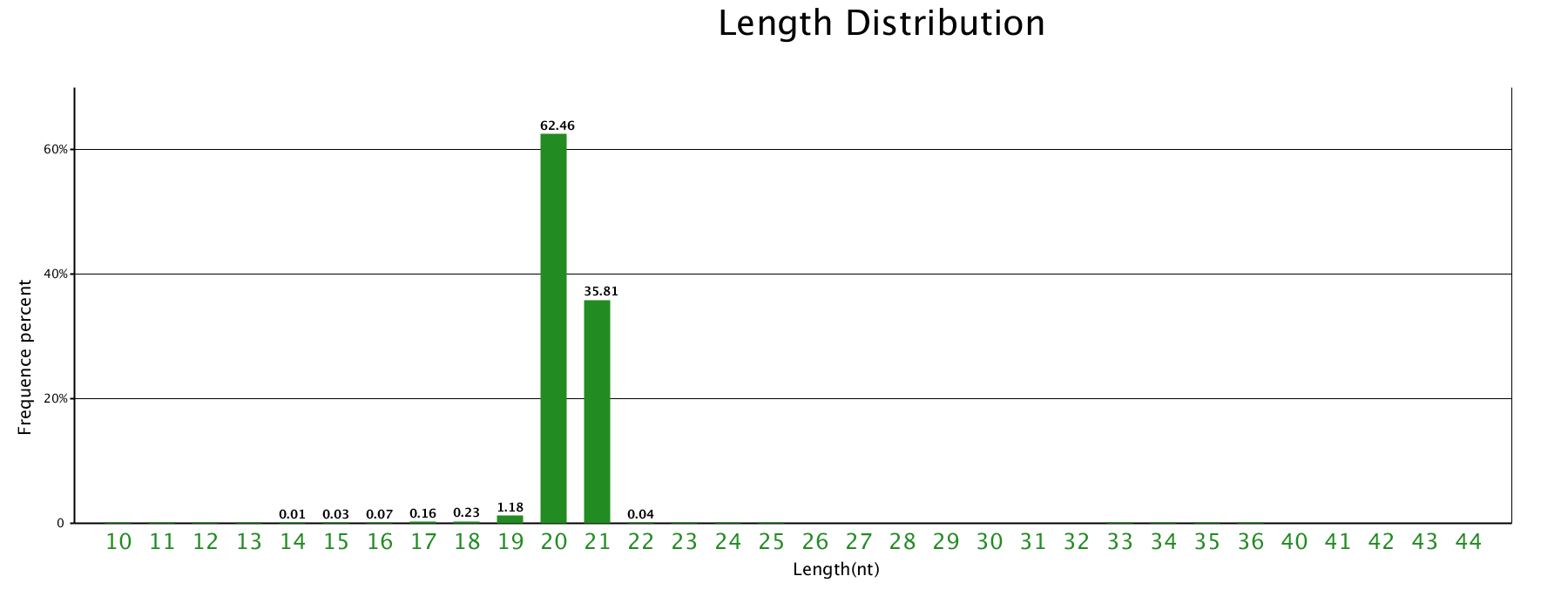

Supplement: Supplementary file 3 — Additional file 3: The length distribution of small RNAs in the Malus hupehensis juvenile leaf degradome library. (DOCX 46 KB) [file 12864_2014_7075_MOESM3_ESM.docx]
