# Supplementary material for: Genome-wide identification of vegetative phase transition-associated microRNAs and target predictions using degradome sequencing in Malus hupehensis
Source: BMC Genomics. 2014 Dec 17;15(1):1125. doi: 10.1186/1471-2164-15-1125 (PMC4523022; doi:10.1186/1471-2164-15-1125)
Supplement: Supplementary file 4 — Additional file 4: Identified known miRNAs in Malus hupehensis and their read counts. (DOCX 42 KB) [file 12864_2014_7075_MOESM4_ESM.docx]

**Additional file 4. Identified known miRNAs in Malus hupehensis and their read counts.**

| **miRNAs** | **Mature miRNA sequence** | **Length(nt)** | **MFE** | **Count** | **Count** | **miRNAs** | **Mature miRNA sequence** | **Length(nt)** | **MFE** | **Count** | **Count** |
| --- | --- | --- | --- | --- | --- | --- | --- | --- | --- | --- | --- |
|  |  |  |  | **(A)** | **(J)** |  |  |  |  | **(A)** | **(J)** |
| mdm-miR1511 | ACCUAGCUCUGAUACCAUGAA | 21 | -45 | 1601 | 1955 | mdm-miR156q | CUGACAGAAGAUAGAGAGCAC | 21 | -45.7 | 157 | 7244 |
| mdm-miR156a | UGACAGAAGAGAGUGAGCAC | 20 | -37.9 | 11912 | 61640 | mdm-miR156r | CUGACAGAAGAUAGAGAGCAC | 21 | -48.9 | 158 | 7244 |
| mdm-miR156aa | UGACAGAAGAUAGAGAGCAC | 20 | -45.7 | 157 | 7244 | mdm-miR156s | CUGACAGAAGAUAGAGAGCAC | 21 | -45.7 | 157 | 7244 |
| mdm-miR156ab | UUGACAGAAGAUAGAGAGCAC | 21 | -82.8 | 14704 | 446198 | mdm-miR156t | UUGACAGAAGAGAGAGAGCAC | 21 | -46.4 | 5367 | 20615 |
| mdm-miR156ac | UUGACAGAAGAUAGAGAGCAC | 21 | -100.7 | 14704 | 446199 | mdm-miR156u | UUGACAGAAGAGAGAGAGCAC | 21 | -45.5 | 5367 | 20615 |
| mdm-miR156ad | UGACAGAAGAAAGUGAGCAC | 20 | -48.2 | 1 | 8 | mdm-miR156v | UUGACAGAAGAGAGAGAGCAC | 21 | -45.5 | 5367 | 20615 |
| mdm-miR156ae | UGACAGAAGAAAGUGAGCAC | 20 | -43.23 | 1 | 8 | mdm-miR156w | UUGACAGAAGAGAGAGAGCAC | 21 | -44.8 | 5367 | 20615 |
| mdm-miR156b | UGACAGAAGAGAGUGAGCAC | 20 | -40 | 7259 | 40353 | mdm-miR156x | UGACAGAAGAUAGAGAGCAC | 20 | -48.9 | 158 | 7244 |
| mdm-miR156c | UGACAGAAGAGAGUGAGCAC | 20 | -50.1 | 11912 | 61640 | mdm-miR156y | UGACAGAAGAUAGAGAGCAC | 20 | -45.7 | 157 | 7244 |
| mdm-miR156d | UGACAGAAGAGAGUGAGCAC | 20 | -62.94 | 7260 | 40359 | mdm-miR156z | UGACAGAAGAUAGAGAGCAC | 20 | -48.9 | 158 | 7244 |
| mdm-miR156e | UGACAGAAGAGAGUGAGCAC | 20 | -40.9 | 11912 | 61640 | mdm-miR159a | CUUGGAUUGAAGGGAGCUCC | 21 | -96.3 | 44 | 69 |
| mdm-miR156f | UGACAGAAGAGAGUGAGCAC | 20 | -77.3 | 11936 | 61710 | mdm-miR159b | CUUGGAUUGAAGGGAGCUCC | 20 | -93.1 | 44 | 69 |
| mdm-miR156g | UGACAGAAGAGAGUGAGCAC | 20 | -43.2 | 7261 | 40358 | mdm-miR159c | GAAUUCCUUCUCCUCUCCUUU | 21 | -96.3 | 0 | 1 |
| mdm-miR156h | UGACAGAAGAGAGUGAGCAC | 20 | -50.1 | 11912 | 61640 | mdm-miR160a | UGCCUGGCUCCCUGUAUGCCA | 21 | -47.6 | 26 | 221 |
| mdm-miR156i | UGACAGAAGAGAGUGAGCAC | 20 | -40.4 | 7260 | 40358 | mdm-miR160b | UGCCUGGCUCCCUGUAUGCCA | 21 | -47 | 26 | 222 |
| mdm-miR156j | UGACAGAAGAGAGUGAGCAC | 20 | -45.3 | 11912 | 61640 | mdm-miR160c | UGCCUGGCUCCCUGUAUGCCA | 21 | -54.7 | 27 | 223 |
| mdm-miR156k | UGACAGAAGAGAGUGAGCAC | 20 | -62 | 11935 | 61695 | mdm-miR160d | UGCCUGGCUCCCUGUAUGCCA | 21 | -47 | 26 | 222 |
| mdm-miR156l | UGACAGAAGAGAGUGAGCAC | 20 | -112.3 | 7260 | 40359 | mdm-miR160e | UGCCUGGCUCCCUGUAUGCCA | 21 | -50.6 | 27 | 223 |
| mdm-miR156m | UGACAGAAGAGAGUGAGCAC | 20 | -55.7 | 7260 | 40358 | mdm-miR162a | UCGAUAAACCUCUGCAUCCAG | 21 | -42.1 | 244 | 514 |
| mdm-miR156n | UGACAGAAGAGAGUGAGCAC | 20 | -65 | 11936 | 61710 | mdm-miR162b | UCGAUAAACCUCUGCAUCCAG | 21 | -43.5 | 244 | 514 |
| mdm-miR156o | UGACAGAAGAGAGUGAGCAC | 20 | -51.5 | 7260 | 40358 | mdm-miR164a | UGGAGAAGCAGGGCACAUGCC | 21 | -42.13 | 0 | 1 |
| mdm-miR156p | CUGACAGAAGAUAGAGAGCAC | 21 | -48.9 | 158 | 7244 | mdm-miR164b | UGGAGAAGCAGGGCACGUGCA | 21 | -55.56 | 4059 | 3500 |
| mdm-miR164c | UGGAGAAGCAGGGCACGUGCA | 21 | -58 | 4055 | 3499 | mdm-miR169a | CAGCCAAGGAUGACUUGCCGG | 21 | -74.1 | 0 | 5 |
| mdm-miR164d | UGGAGAAGCAGGGCACGUGCA | 21 | -65.9 | 4056 | 3499 | mdm-miR169b | UAGCCAAGGAUGAUUUGCCUGC | 22 | -70.61 | 1 | 7 |
| mdm-miR164e | UGGAGAAGCAGGGCACGUGCA | 21 | -53.03 | 4069 | 3490 | mdm-miR169c | UAGCCAAGGAUGACUUGCCCG | 21 | -68.23 | 0 | 0 |
| mdm-miR164f | UGGAGAAGCAGGGCACGUGCA | 21 | -64.41 | 4069 | 3490 | mdm-miR169d | UAGCCAAGGAUGACUUGCCCG | 21 | -87.45 | 0 | 0 |
| mdm-miR166a | UCGGACCAGGCUUCAUUCCCC | 21 | -69 | 318332 | 312466 | mdm-miR169e | UGAAGAGAAGAGCGUUGUUUGG | 22 | -84.76 | 1 | 8 |
| mdm-miR166b | UCGGACCAGGCUUCAUUCCCC | 21 | -45.6 | 318381 | 312528 | mdm-miR169f | UGAAGAGAAGAGCGUUGUUUGG | 22 | -84.76 | 1 | 8 |
| mdm-miR166c | UCGGACCAGGCUUCAUUCCCC | 21 | -50.69 | 318528 | 312586 | mdm-miR171a | UUGAGCCGCGUCAAUAUCUCC | 21 | -37.1 | 303 | 431 |
| mdm-miR166d | UCGGACCAGGCUUCAUUCCCC | 21 | -63.76 | 318388 | 312508 | mdm-miR171b | UUGAGCCGCGUCAAUAUCUCC | 21 | -45.2 | 312 | 439 |
| mdm-miR166e | UCGGACCAGGCUUCAUUCCCC | 21 | -50.8 | 318768 | 312715 | mdm-miR171c | UGAUUGAGCCGCGCCAAUAUC | 21 | -54.6 | 85 | 66 |
| mdm-miR166f | UCGGACCAGGCUUCAUUCCCC | 21 | -47.2 | 318618 | 312654 | mdm-miR171d | UGAUUGAGCCGCGCCAAUAUC | 21 | -39.2 | 85 | 66 |
| mdm-miR166g | UCGGACCAGGCUUCAUUCCCC | 21 | -74.6 | 318332 | 312466 | mdm-miR171e | UGAUUGAGCCGCGCCAAUAUC | 21 | -62.4 | 85 | 66 |
| mdm-miR166h | UCGGACCAGGCUUCAUUCCCC | 21 | -66.36 | 318332 | 312467 | mdm-miR171f | UUGAGCCGUGCCAAUAUCACG | 21 | -56.98 | 0 | 3 |
| mdm-miR166i | UCGGACCAGGCUUCAUUCCCC | 21 | -50.8 | 318768 | 312715 | mdm-miR171g | UGAUUGAGCCGUGCCAAUAUC | 21 | -49.43 | 0 | 0 |
| mdm-miR167a | AGAUCAUCUGGCAGUUUCACC | 21 | -57.3 | 8 | 7 | mdm-miR171h | UGAUUGAGCCGUGCCAAUAUC | 21 | -55.34 | 0 | 0 |
| mdm-miR167b | UGAAGCUGCCAGCAUGAUCUA | 21 | -51.2 | 80031 | 129341 | mdm-miR171i | UGAGCCGAACCAAUAUCACUC | 21 | -55 | 1 | 0 |
| mdm-miR167c | UGAAGCUGCCAGCAUGAUCUA | 21 | -39.5 | 105736 | 166808 | mdm-miR171j | UUGAGCCGCGCCAAUAUCACU | 21 | -37.8 | 7 | 9 |
| mdm-miR167d | UGAAGCUGCCAGCAUGAUCUA | 21 | -50.8 | 105736 | 166808 | mdm-miR171k | UUGAGCCGCGCCAAUAUCACU | 21 | -45.9 | 9 | 9 |
| mdm-miR167e | UGAAGCUGCCAGCAUGAUCUA | 21 | -42.82 | 80031 | 129341 | mdm-miR171l | UUGAGCCGCGCCAAUAUCACU | 21 | -45.9 | 9 | 9 |
| mdm-miR167f | UGAAGCUGCCAGCAUGAUCUA | 21 | -80.1 | 105716 | 166824 | mdm-miR171m | UUGAGCCGUGCCAAUAUCACA | 22 | -41.4 | 1 | 3 |
| mdm-miR167g | UGAAGCUGCCAGCAUGAUCUA | 21 | -67.65 | 105776 | 166874 | mdm-miR171n | UUGAGCCGUGCCAAUAUCACA | 21 | -39 | 1 | 3 |
| mdm-miR167h | UGAAGCUGCCAGCAUGAUCUUA | 22 | -57.3 | 13048 | 13912 | mdm-miR171o | UGGGAUGUUGGUAUGGUUCAA | 21 | -39 | 2 | 0 |
| mdm-miR167i | UGAAGCUGCCAGCAUGAUCUUA | 22 | -57.3 | 13048 | 13912 | mdm-miR172a | AGAAUCUUGAUGAUGCUGCA | 20 | -40.72 | 70 | 42 |
| mdm-miR167j | UGAAGCUGCCAGCAUGAUCUUA | 22 | -59.2 | 13048 | 13912 | mdm-miR172b | AGAAUCUUGAUGAUGCUGCA | 20 | -49.3 | 70 | 42 |
| mdm-miR168a | UCGCUUGGUGCAGGUCGGGAA | 21 | -76.5 | 10484 | 13108 | mdm-miR172c | AGAAUCUUGAUGAUGCUGCA | 20 | -49.12 | 70 | 42 |
| mdm-miR168b | UCGCUUGGUGCAGGUCGGGAA | 21 | -102.1 | 10484 | 13108 | mdm-miR172d | AGAAUCUUGAUGAUGCUGCAU | 21 | -63.6 | 26552 | 12319 |
| mdm-miR172e | AGAAUCUUGAUGAUGCUGCAU | 21 | -58.1 | 26552 | 12319 | mdm-miR390d | AAGCUCAGGAGGGAUAGCGCC | 21 | -51.4 | 38 | 50 |
| mdm-miR172f | AGAAUCUUGAUGAUGCUGCAU | 21 | -63.6 | 26552 | 12319 | mdm-miR390e | AAGCUCAGGAGGGAUAGCGCC | 21 | -47.7 | 34 | 51 |
| mdm-miR172g | AGAAUCUUGAUGAUGCUGCAU | 21 | -51.8 | 26576 | 12330 | mdm-miR390f | AAGCUCAGGAGGGAUAGCGCC | 21 | -47.7 | 34 | 51 |
| mdm-miR172h | AGAAUCUUGAUGAUGCUGCAU | 21 | -51.8 | 26576 | 12330 | mdm-miR391 | UACGCAGGAGAGAUGACGCCG | 21 | -53.2 | 44526 | 60184 |
| mdm-miR172i | GGAAUCUUGAUGAUGCUGCAU | 21 | -75.5 | 59 | 19 | mdm-miR393a | UCCAAAGGGAUCGCAUUGAUCU | 22 | -57.3 | 42 | 77 |
| mdm-miR172j | GGAAUCUUGAUGAUGCUGCAU | 21 | -75.5 | 59 | 19 | mdm-miR393b | UCCAAAGGGAUCGCAUUGAUCU | 22 | -63.6 | 42 | 77 |
| mdm-miR172k | GGAAUCUUGAUGAUGCUGCAU | 21 | -75.5 | 59 | 19 | mdm-miR393c | UCCAAAGGGAUCGCAUUGAUCU | 22 | -63.6 | 42 | 77 |
| mdm-miR172l | GGAAUCUUGAUGAUGCUGCAG | 21 | -74.1 | 0 | 0 | mdm-miR393d | AUCAUGCGAUCCCUUCGGACG | 21 | -63.6 | 42 | 75 |
| mdm-miR172m | AGAAUCUUGAUGAUGCUGCAG | 21 | -40.72 | 70 | 42 | mdm-miR393e | AUCAUGCGAUCCCUUCGGACG | 21 | -63.6 | 42 | 75 |
| mdm-miR172n | AGAAUCUUGAUGAUGCUGCAG | 21 | -49.12 | 70 | 42 | mdm-miR393f | AUCAUGCGAUCCCUUCGGACG | 21 | -57.3 | 42 | 75 |
| mdm-miR172o | AGAAUCUUGAUGAUGCUGCAG | 21 | -49.3 | 70 | 42 | mdm-miR394a | UUGGCAUUCUGUCCACCUCC | 20 | -80.3 | 2 | 13 |
| mdm-miR2111a | UAAUCUGCAUCCUGAGGUUUA | 21 | -56.3 | 14 | 9 | mdm-miR394b | UUGGCAUUCUGUCCACCUCC | 20 | -89.1 | 2 | 12 |
| mdm-miR2111b | UAAUCUGCAUCCUGAGGUUUA | 21 | -58.6 | 14 | 9 | mdm-miR395a | CUGAAGUGUUUGGGGGAACUC | 21 | -46.2 | 34 | 19 |
| mdm-miR2118a | CUACCGAUGCCACUAAGUCCCA | 22 | -78.7 | 3247 | 6599 | mdm-miR395b | CUGAAGUGUUUGGGGGAACUC | 21 | -62.9 | 34 | 19 |
| mdm-miR2118b | CUACCGAUGCCACUAAGUCCCA | 22 | -50.4 | 3247 | 6599 | mdm-miR395c | CUGAAGUGUUUGGGGGAACUC | 21 | -60.8 | 34 | 19 |
| mdm-miR2118c | CUACCGAUGCCACUAAGUCCCA | 22 | -50.7 | 3247 | 6599 | mdm-miR395d | CUGAAGUGUUUGGGGGAACUC | 21 | -55.4 | 34 | 19 |
| mdm-miR319a | UUGGACUGAAGGGAGCUCCCU | 21 | -59.33 | 0 | 8 | mdm-miR395e | CUGAAGUGUUUGGGGGAACUC | 21 | -60.8 | 34 | 19 |
| mdm-miR319b | UUGGACUGAAGGGAGCUCCCU | 21 | -54.22 | 0 | 8 | mdm-miR395f | CUGAAGUGUUUGGGGGAACUC | 21 | -60.8 | 34 | 19 |
| mdm-miR319c | AUCCAACGAAGCAGGAGCUGA | 21 | -89.77 | 0 | 12 | mdm-miR395g | CUGAAGUGUUUGGGGGAACUC | 21 | -57.46 | 34 | 19 |
| mdm-miR3627a | UCGCAGGAGAGAUGGCACUA | 20 | -38.6 | 83 | 191 | mdm-miR395h | CUGAAGUGUUUGGGGGAACUC | 21 | -46.1 | 34 | 19 |
| mdm-miR3627b | UCGCAGGAGAGAUGGCACUA | 20 | -36.92 | 83 | 191 | mdm-miR395i | CUGAAGUGUUUGGGGGAACUC | 21 | -50.3 | 34 | 19 |
| mdm-miR3627c | UCGCAGGAGAGAUGGCACUA | 20 | -50.8 | 83 | 191 | mdm-miR396a | UUCCACAGCUUUCUUGAACAG | 21 | -62.5 | 925 | 1648 |
| mdm-miR390a | AAGCUCAGGAGGGAUAGCGCC | 21 | -61.8 | 35 | 50 | mdm-miR396b | UUCCACAGCUUUCUUGAACUG | 21 | -66.3 | 220 | 747 |
| mdm-miR390b | AAGCUCAGGAGGGAUAGCGCC | 21 | -51.1 | 34 | 51 | mdm-miR396c | UUCCACAGCUUUCUUGAACUU | 21 | -54.6 | 62 | 94 |
| mdm-miR390c | AAGCUCAGGAGGGAUAGCGCC | 21 | -55.1 | 38 | 50 | mdm-miR396d | UUCCACAGCUUUCUUGAACUU | 21 | -50.6 | 62 | 94 |
| mdm-miR396e | UUCCACAGCUUUCUUGAACUU | 21 | -54.6 | 62 | 94 | mdm-miR477 | ACUCUCCCUCAAGAGCUUCUC | 21 | -55.42 | 0 | 2 |
| mdm-miR396f | UUCCACGGCUUUCUUGAACUG | 21 | -53.4 | 281 | 296 | mdm-miR477b | ACUCUCCCUCAAGGGCUUCGAC | 22 | -49.42 | 0 | 5 |
| mdm-miR396g | UUCCACGGCUUUCUUGAACUG | 21 | -52.3 | 281 | 295 | mdm-miR482a-3p | UUCCCAAGCCCGCCCAUUCCUA | 22 | -61.20 | 5649 | 5069 |
| mdm-miR397a | UUGAGUGCAGCGUUGAUGAAA | 21 | -41.7 | 72 | 22 | mdm-miR482a-5p | AGGAAUGGGCUGUUUGGGAAGA | 22 | -48.8 | 121128 | 109535 |
| mdm-miR397b | UUGAGUGCAGCGUUGAUGAAA | 21 | -55.2 | 72 | 22 | mdm-miR482b | UCUUUCCUAUCCCUCCCAUUCC | 22 | -45.9 | 61 | 29 |
| mdm-miR398a | UGUGUUCUCAGGUCACCCCUU | 21 | -58.4 | 25 | 10 | mdm-miR482c | UCUUUCCUAACCCUCCCAUUCC | 22 | -54.66 | 59 | 33 |
| mdm-miR398b | UGUGUUCUCAGGUCGCCCCUG | 21 | -55.82 | 228 | 32 | mdm-miR482d | AAUGGAAGGGUAGGAAAGAAG | 21 | -45.9 | 9202 | 6231 |
| mdm-miR398c | UGUGUUCUCAGGUCGCCCCUG | 21 | -66 | 228 | 32 | mdm-miR5225a | UCUGUCGAAGGUGAGAUGGUGC | 22 | -62.11 | 103 | 1026 |
| mdm-miR399a | UGCCAAAGGAGAAUUGCCCUG | 21 | -55.4 | 6 | 0 | mdm-miR5225b | UCUGUCGAAGGUGAGAUGGUGC | 22 | -62.11 | 103 | 1026 |
| mdm-miR399b | UGCCAAAGGAGAAUUGCCCUG | 21 | -49.4 | 6 | 0 | mdm-miR5225c | UCUGUCGUGGGUGAGAUGGUGC | 22 | -51.3 | 205 | 191 |
| mdm-miR399c | UGCCAAAGGAGAAUUGCCCUG | 21 | -45.4 | 6 | 0 | mdm-miR535a | UGACAACGAGAGAGAGCACGC | 21 | -48 | 1580 | 46260 |
| mdm-miR399d | UGCCAAAGGAGAGUUGCCCUA | 21 | -68.5 | 1 | 3 | mdm-miR535b | UGACAAGGAGAGAGAGCACGC | 21 | -61.4 | 241 | 9540 |
| mdm-miR399e | UGCCAAAGGAGAUUUGCUCGG | 21 | -49.55 | 0 | 0 | mdm-miR535c | UGACAAGGAGAGAGAGCACGC | 21 | -55.4 | 240 | 9536 |
| mdm-miR399f | UGCCAAAGGAGAUUUGCUCGG | 21 | -49.33 | 0 | 0 | mdm-miR535d | UGACGACGAGAGAGAGCACGC | 21 | -55.7 | 22652 | 188682 |
| mdm-miR399g | UGCCAAAGGAGAUUUGCUCGG | 21 | -65.43 | 0 | 0 | mdm-miR7120a | UGUUAUAUUGUCAGAUUGUCA | 21 | -101.2 | 6547 | 12581 |
| mdm-miR399h | UGCCAAAGGAGAUUUGCUCGG | 21 | -58.43 | 0 | 0 | mdm-miR7120b | UGUUAUAUUGUCAGAUUGUCA | 21 | -101.2 | 6547 | 12581 |
| mdm-miR399i | UGCCAAAGGAGAGUUGCCCUG | 21 | -46.8 | 8 | 1 | mdm-miR7121a | UCCUCUUGGUGAUCGCCCUGU | 21 | -50.9 | 6320 | 9353 |
| mdm-miR399j | UGCCAAAGGAGAGUUGCCCUG | 21 | -50.6 | 8 | 1 | mdm-miR7121b | UCCUCUUGGUGAUCGCCCUGU | 21 | -72.7 | 6316 | 9345 |
| mdm-miR403a | UUAGAUUCACGCACAAACUCG | 21 | -44.29 | 162 | 254 | mdm-miR7121c | UCCUCUUGGUGAUCGCCCUGU | 21 | -72.2 | 6304 | 9316 |
| mdm-miR403b | UUAGAUUCACGCACAAACUCG | 21 | -42.97 | 162 | 254 | mdm-miR7121d | UCCUCUUGGUGAUCGCCCUGC | 21 | -67.8 | 23 | 38 |
| mdm-miR408a | AUGCACUGCCUCUUCCCUGGC | 21 | -70.1 | 1787 | 987 | mdm-miR7121e | UCCUCUUGGUGAUCGCCCUGC | 21 | -67.8 | 23 | 38 |
| mdm-miR408b | ACAGGGAAGAGGUAGAGCAUG | 21 | -58.5 | 39392 | 4902 | mdm-miR7121f | UCCUCUUGGUGAUCGCCCUGC | 21 | -40.01 | 19 | 31 |
| mdm-miR408c | ACAGGGAAGAGGUAGAGCAUG | 21 | -69 | 39843 | 4977 | mdm-miR7121g | UCCUCUUGGUGAUCGCCCUGC | 21 | -46.5 | 632 | 768 |
| mdm-miR408d | ACAGGGAAGAGGUAGAGCAUG | 21 | -58.5 | 39392 | 4902 | mdm-miR7121h | UCCUCUUGGUGAUCGCCCUGC | 21 | -67.8 | 23 | 38 |
| mdm-miR397a | UUGAGUGCAGCGUUGAUGAAA | 21 | -41.7 | 72 | 22 | mdm-miR7122a | UUAUACAGAGAAAUCACGGUCG | 22 | -45.33 | 2173 | 3160 |
| mdm-miR7122b | UUAUACAGAGAAAUCACGGUCG | 22 | *-45.33* | *2173* | 3160 | mdm-miR7127a | AUACUCAUCGAAUUUGUCAUA | 21 | *-56.35* | *1* | 0 |
| mdm-miR7123a | AAGAGCGGGAUGUGUAAAAGG | 21 | *-69.9* | *100* | 92 | mdm-miR7127b | AUACUCAUCGAAUUUGUCAUA | 21 | *-53.25* | *1* | 0 |
| mdm-miR7123b | AAGAGCGGGAUGUGUAAAAGG | 21 | *-69.9* | *100* | 92 | mdm-miR7128 | AUCAUUAACACUUAAUAACGA | 21 |  | *0* | 0 |
| mdm-miR7124a | CACCAAUAUCAACUUUAUUUG | 21 | *-86.8* | *29* | 58 | mdm-miR827 | UUAGAUGACCAUCAACGAACA | 21 | *-66* | *2* | 1 |
| mdm-miR7124b | CACCAAUAUCAACUUUAUUUG | 21 | *-109* | *29* | 58 | mdm-miR828a | UCUUGCUCAAAUGAGUAUUCCA | 22 | *-66.7* | *0* | 1 |
| mdm-miR7125 | CGAACUUAUUGCAACUAGCUU | 21 | *-43.1* | *1896* | 603 | mdm-miR828b | UCUUGCUCAAAUGAGUAUUCCA | 22 | *-73.4* | *0* | 1 |
| mdm-miR7126 | AAAGUAUCAAGGAGCGCAAAG | 21 | *-67.7* | *35* | 41 | mdm-miR858 | UUCGUUGUCUGUUCGACCUGA | 21 | *-245.59* | *238* | 404 |
