# Supplementary material for: Genome-wide identification of vegetative phase transition-associated microRNAs and target predictions using degradome sequencing in Malus hupehensis
Source: BMC Genomics. 2014 Dec 17;15(1):1125. doi: 10.1186/1471-2164-15-1125 (PMC4523022; doi:10.1186/1471-2164-15-1125)
Supplement: Supplementary file 5 — Additional file 5: Identified novel miRNAs in Malus hupehensis and their read counts. (DOCX 46 KB) [file 12864_2014_7075_MOESM5_ESM.docx]

**Additional file 5. Identified novel miRNAs in *Malus hupehensis* and their read counts.**

| Name | Length (nt) | miRNA sequence | A (count) | J (count) |
| --- | --- | --- | --- | --- |
| novel_mir_10 | 23 | AAUGGGCUAGCAAUAAUGUGGUA | 16 | 14 |
| novel_mir_100 | 21 | UUCGAUUGGGAAACAGAUGGA | 63 | 98 |
| novel_mir_102 | 21 | AUUUUUURUGUGUUUUCGUCU | 175 | 282 |
| novel_mir_103 | 23 | UUUGUAACCGUUGGAUCAAAAUC | 18 | 11 |
| novel_mir_106 | 21 | UCAAAUUUCAAUGGUUCGGAU | 49 | 42 |
| novel_mir_108 | 22 | CUGCAAUGUCGGGGUCUUCAUG | 70 | 60 |
| novel_mir_11 | 21 | UGAAGUUUUAGCACRAUGGUU | 34 | 0 |
| novel_mir_114 | 21 | AGAGAUAGGAGAAAUGUGGAA | 57 | 33 |
| novel_mir_115 | 23 | AAAUUAGGUGUAGAAAGAGGUUA | 10 | 19 |
| novel_mir_116 | 21 | UUGUCGCAKGAGAGAUGGUAC | 1459 | 5562 |
| novel_mir_118 | 23 | UAGUAUAAGUGGGAGCCUCGCGG | 23 | 0 |
| novel_mir_119 | 21 | AAAGUAAAGAAUGAAGGCGUG | 7 | 14 |
| novel_mir_120 | 21 | AGGCAAUCAUGUAGUAUCCCC | 11 | 15 |
| novel_mir_123 | 22 | UCAACUUCUACAGGUACGCAUC | 26 | 127 |
| novel_mir_124 | 21 | UUAGUGGUAAUUUGGUCAGGU | 17 | 20 |
| novel_mir_127 | 23 | AUGGAUAAUGCUCGUGGUCAAUA | 17 | 0 |
| novel_mir_128 | 21 | AGGUGCAGGUGCCAGUGCAGG | 521 | 462 |
| novel_mir_13 | 21 | UUUUAUGAUUGUGAAAUGGAG | 40 | 45 |
| novel_mir_130 | 23 | AAUUGACGUUAAACUGUGAGGUA | 498 | 406 |
| novel_mir_133 | 21 | UUUCGGAUGAAGAUUGCCCAU | 339 | 467 |
| novel_mir_136 | 23 | AAGUUGGAUGUAGAGAUAGGUUA | 96 | 100 |
| novel_mir_137 | 23 | UUAGGGAUAAGAUUGGAUUGACA | 20 | 18 |
| novel_mir_139 | 23 | AAAUUGAGUGUAGAAAGAGGUUA | 85 | 0 |
| novel_mir_144 | 23 | AGCAGGACCGGCUGCUGAAUUUA | 17 | 0 |
| novel_mir_148 | 23 | UGUUGAAAUUUGAUCCAARGGUU | 23 | 20 |
| novel_mir_149 | 21 | UUGCCUUGCUUGAACCGACGA | 182 | 352 |
| novel_mir_15 | 21 | UGGACAAAACUUGGAAGUUGG | 32 | 63 |
| novel_mir_150 | 21 | AAUCGUACGGACGACGACGAU | 12 | 15 |
| novel_mir_151 | 21 | AUGAGCUUCGGCGGUGACUGG | 17 | 32 |
| novel_mir_153 | 23 | GCAGGAUGUGURGUGAGUGGCUA | 43 | 0 |
| novel_mir_154 | 23 | GCAGGAUGUGUGGUGAGUGGCUA | 43 | 0 |
| novel_mir_156 | 22 | CUGCCAAAGGAGAUCUGCUCAG | 19 | 0 |
| novel_mir_158 | 21 | AGGACAAAGCUGCAUAUUUAG | 11 | 25 |
| novel_mir_16 | 23 | AAAGUGAUUGUGUAUAAAUAGGU | 33 | 28 |
| novel_mir_160 | 21 | UAUGCAGGAGAGAUGACGCCG | 76 | 34 |
| novel_mir_165 | 23 | GCUGGACUCCUCAAUGCACGGUU | 56 | 31 |
| novel_mir_17 | 22 | UCACCAUUGCAUCUCAUGUUCC | 565 | 338 |
| novel_mir_173 | 20 | AACUGAGAUGCUAAGGUGAU | 85 | 0 |
| novel_mir_174 | 22 | UUGGAAAACCUAUACGCUCACA | 27 | 45 |
| novel_mir_177 | 21 | UAAAGUUGUAUUGUAAUGCGG | 28 | 0 |
| novel_mir_178 | 23 | AAAUUGGGUGUAGAAAGAGGUUA | 18 | 16 |
| novel_mir_18 | 21 | UUGCUCGCGAUCGAGGACAGG | 22 | 33 |
| novel_mir_181 | 21 | CAAGGUGGUGGGACAUCGGUU | 21 | 0 |
| novel_mir_182 | 21 | AUUGUAGCAGAUGCACACAUA | 30 | 19 |
| novel_mir_185 | 21 | CGAACUGGAUUGUUUGACACG | 19 | 20 |
| novel_mir_188 | 23 | ACAUGAUUAGUUUGGAAUACCGU | 30 | 30 |
| novel_mir_194 | 21 | UCCGAAGGCUGGGAUCCGGAG | 8 | 15 |
| novel_mir_2 | 21 | UAGAUAGGUGGUACAAAUGUG | 108 | 142 |
| novel_mir_204 | 23 | AUGGUUUUUCCGGCGGCAGGACA | 27 | 0 |
| novel_mir_205 | 23 | GUUGAAUUUUGAUCCAACGGCUA | 45 | 0 |
| novel_mir_207 | 21 | UUUCGGAGGAAGAAGAAGGAA | 52 | 110 |
| novel_mir_212 | 23 | UGUUUGUAAUCGUUGGAUCAAAU | 25 | 0 |
| novel_mir_213 | 22 | UGAGGUCAGUGAGGUACAGUUG | 28 | 0 |
| novel_mir_219 | 23 | ACACGGAUGGUACACCACAUGUU | 24 | 0 |
| novel_mir_22 | 21 | UAGGCGUAGAUAGACCGUGGG | 7929 | 10433 |
| novel_mir_223 | 21 | UUUGAAGGAGAAAUGGAUGAC | 40 | 94 |
| novel_mir_232 | 22 | UGGUUUUCUUCCUGUUCGUCAU | 8 | 17 |
| novel_mir_233 | 23 | UUUGAUCCAACGGCUAAAAUUAU | 24 | 0 |
| novel_mir_237 | 23 | AGGACACCGGAAGUAGGAGGGCA | 123 | 0 |
| novel_mir_241 | 23 | UGUUCAUCAUACAUCGUGGGUCA | 26 | 0 |
| novel_mir_242 | 23 | AUGGGAACACAAGUGGUACAUCA | 24 | 0 |
| novel_mir_244 | 23 | AGUAAGGCUGCGUACGACAAACA | 104 | 57 |
| novel_mir_246 | 23 | UGUUUGUAGACGUUGGAUCAAAU | 19 | 14 |
| novel_mir_249 | 23 | AAUGUAUGGGAAGUGGGAGGGUA | 29 | 7 |
| novel_mir_250 | 23 | CUCUGUUUGUAGCCGUUGGAUCA | 90 | 0 |
| novel_mir_251 | 21 | AACCUACAGAAACACUACCAC | 24 | 0 |
| novel_mir_253 | 21 | UAACRAUGAUUGUCAAUUGGA | 130 | 182 |
| novel_mir_254 | 21 | UCAAGUGAUUCGGAUCGUUAA | 56 | 0 |
| novel_mir_257 | 21 | UCAAGUAGUUAAGAGCAUUUA | 16 | 40 |
| novel_mir_260 | 23 | ACGGGAACACAAGUGGUACAUCA | 244 | 151 |
| novel_mir_262 | 20 | UAGCCAGGGAUGACUUGCCU | 6 | 100 |
| novel_mir_263 | 23 | UGAAAAUUGAUCUAACGACUACA | 31 | 30 |
| novel_mir_267 | 23 | UAAGAGGGUGGGAGAGUGGGCUA | 79 | 17 |
| novel_mir_268 | 21 | UGAGCCAAGGAUGACUUGCCA | 8 | 61 |
| novel_mir_269 | 23 | AUGUGGGACUCACAUGUAUUAGA | 93 | 0 |
| novel_mir_27 | 23 | AGGGCACUGAAAGUGGAAGGACA | 33 | 12 |
| novel_mir_273 | 21 | UUGGUGAUCGUCAAAGCCUUU | 139 | 141 |
| novel_mir_275 | 23 | ACGGAACGGGACGGGACGGGACA | 1835 | 0 |
| novel_mir_276 | 21 | UUGAGGAUGCAUAGUUUUCAG | 7343 | 7927 |
| novel_mir_282 | 21 | AACAUUAUUCGGCCGUUAUUU | 32 | 0 |
| novel_mir_284 | 21 | CUUUGGAUAGAGCAAGGCGUC | 18 | 31 |
| novel_mir_290 | 23 | ACGGGACGGGACGGGACGGGACA | 597 | 263 |
| novel_mir_291 | 23 | AUCAAAUUUUAAUGGUCCGGAUA | 32 | 0 |
| novel_mir_292 | 23 | AGAACUUCUGCAGAACCAGGUCA | 68 | 26 |
| novel_mir_294 | 23 | AAGUGACAUAAUAGAAUUGGUUA | 19 | 0 |
| novel_mir_296 | 23 | UCGUGUACUGUCAUGUGGGGAUG | 21 | 0 |
| novel_mir_30 | 23 | AGCGAGUAUGUCGACUGAUGUCA | 112 | 53 |
| novel_mir_300 | 21 | ACAUGCAAUGAGAUUUGUGGA | 22 | 15 |
| novel_mir_302 | 21 | AAGAAACGGCGUACCUUUCUA | 1039 | 1877 |
| novel_mir_310 | 21 | ACAGGUUGAAAUAUCAUUCUU | 30 | 32 |
| novel_mir_312 | 23 | GAUGGAAACACGGUAACUCAUAA | 33 | 0 |
| novel_mir_315 | 22 | RUGCCAAAGGAGAUCUGCUCAG | 19 | 0 |
| novel_mir_316 | 22 | UGACCUUGCAUUCUUGAUGACC | 21 | 0 |
| novel_mir_319 | 21 | UUUGCUGGUUUUUUGGAUAUU | 76 | 0 |
| novel_mir_320 | 21 | ACAAAAGAAACGUUGGAUGAU | 264 | 545 |
| novel_mir_321 | 21 | CUAGCGAAUCACGGAUGGACA | 18 | 0 |
| novel_mir_322 | 23 | UAAGUUGAUUUUUGGACGUGCUA | 20 | 0 |
| novel_mir_324 | 23 | UCUUGAAAUUUGAUCCAACGGUU | 18 | 0 |
| novel_mir_325 | 21 | UUGAAUAAAAAAUGAACGGCC | 25 | 67 |
| novel_mir_326 | 23 | UCGUGGUUUGUGGGAGAUGUCUA | 37 | 12 |
| novel_mir_329 | 20 | GAGGACGAGGAAGAUGGGGA | 12 | 35 |
| novel_mir_333 | 22 | UAAAUGGAUGGUAUAAAUGCAU | 5 | 16 |
| novel_mir_334 | 21 | GGUAUACAAAGUCAACAGAUC | 139 | 162 |
| novel_mir_336 | 23 | AAGCUGGGUGUAGAGAUAGGUUA | 36 | 0 |
| novel_mir_338 | 22 | CUUGAAGAAGACGACUCGUAGA | 22 | 0 |
| novel_mir_340 | 21 | UUUUAAAAUGAUUGAGAGCGC | 17 | 14 |
| novel_mir_341 | 21 | CUUCUACUUUUGUCUGACUUU | 25 | 0 |
| novel_mir_343 | 21 | CUGAUGUAGAGAAAGUGUUGA | 488 | 745 |
| novel_mir_348 | 21 | UCUGAAGCAACUCAAGAGCCU | 14 | 21 |
| novel_mir_353 | 22 | UGAAGAAUCAUGAUGUUGCAUU | 0 | 22 |
| novel_mir_355 | 22 | UAUCCCAUGGCAGCUCAAGGCA | 0 | 16 |
| novel_mir_363 | 21 | AAGUAUUAUGGACUUUUGGCA | 0 | 19 |
| novel_mir_364 | 23 | AAUACAAAUAGUUGGAUACUUGA | 0 | 22 |
| novel_mir_367 | 23 | UGGAUAUGUGUGUUAAAAGGUUA | 0 | 20 |
| novel_mir_374 | 21 | UAGUGGAGGAAAUGUUGCAUG | 0 | 15 |
| novel_mir_38 | 22 | AAGUAUUAUGGACUUUUGGCAC | 20 | 0 |
| novel_mir_383 | 21 | UAGAGAUGUGAUACAAAUGUG | 0 | 62 |
| novel_mir_388 | 21 | UCUUGAAACAGAAGCUGGACG | 0 | 119 |
| novel_mir_390 | 23 | UUUUGAUCCAACGGCUACAAAUA | 0 | 30 |
| novel_mir_392 | 21 | UAGCCAAGGAUGACUUGCCUA | 0 | 15 |
| novel_mir_395 | 21 | UUCGGAAGUUGAUUGAUUGGA | 0 | 54 |
| novel_mir_396 | 23 | ACGGAACGAAGGUGUAAUUUUUA | 0 | 32 |
| novel_mir_4 | 21 | UUGGUUUGCAUUGUUGAGAUA | 46 | 84 |
| novel_mir_40 | 23 | AAACACGGUCUGAUACAUCAAGU | 25 | 0 |
| novel_mir_402 | 21 | UUUGCGGAUAGAGAUAACGGA | 0 | 156 |
| novel_mir_404 | 21 | UAUGAUCAUGAAAGUAGGUAU | 0 | 17 |
| novel_mir_409 | 20 | UAGUAUAAGUGGGAGCCUCG | 0 | 29 |
| novel_mir_41 | 21 | AAGGGAUUGAGAAGGUAAUAU | 20 | 0 |
| novel_mir_416 | 21 | UUACCGGUAAGAACACGUGCA | 0 | 54 |
| novel_mir_419 | 21 | UCCCCGUCAUCGUCGUUCGUA | 0 | 14 |
| novel_mir_424 | 23 | GAURAAAUUUUAACGGUCRGGAU | 0 | 16 |
| novel_mir_426 | 20 | UUGGAUAAAAAUUGAACGGC | 0 | 43 |
| novel_mir_429 | 23 | UCUUGAAAUGAGAAAGUCUGAUA | 0 | 67 |
| novel_mir_44 | 23 | GGUGAAUCGCACGAUGGUGGCCA | 26 | 0 |
| novel_mir_440 | 21 | UCAAAUUUUAAGAGUCUGGAU | 0 | 52 |
| novel_mir_447 | 21 | UGAAAUUUUAAAGGUCCGGAU | 0 | 19 |
| novel_mir_448 | 23 | AUGGCUAGCAAUGAUGUGGUUCA | 0 | 15 |
| novel_mir_455 | 21 | GGACACCGGAAGUAGGAGGGC | 0 | 59 |
| novel_mir_459 | 23 | UAGUAAAAAUUAGGGACCGGAUA | 0 | 14 |
| novel_mir_462 | 20 | UAAGUGAUGGUACAACUAGA | 0 | 15 |
| novel_mir_466 | 21 | AGGUAUUGGCGCGCCUCAAUU | 0 | 19 |
| novel_mir_467 | 23 | AGGAGAGCAACUUACAUGAAACA | 0 | 31 |
| novel_mir_47 | 22 | UUGAGAAUAUGGAAUAGUCGGU | 19 | 0 |
| novel_mir_477 | 21 | UGAUGAAGAAGAAGCUGCUAA | 0 | 22 |
| novel_mir_479 | 21 | UAGGGCAAGAAGAAUAGCACA | 0 | 258 |
| novel_mir_48 | 23 | GUGGCCUUGGUGGAAGAGAUCUA | 19 | 11 |
| novel_mir_484 | 21 | UUAACCUCAAAAGUGUCGGAU | 0 | 29 |
| novel_mir_485 | 21 | CGGRUCCCGCCUUGCAUCAAC | 0 | 211 |
| novel_mir_486 | 21 | UGCAUUUGCACCUGCACUUGU | 0 | 198 |
| novel_mir_489 | 22 | CAGAUUGUAAAACAUGGUGGCA | 0 | 14 |
| novel_mir_490 | 23 | AGGUAGAACCAUACACUUAUAUA | 0 | 14 |
| novel_mir_492 | 21 | CGUGGCAUCAUCAAGAUUCAC | 0 | 16 |
| novel_mir_495 | 21 | UUUUGAAGGGAGAAAUGGAUG | 0 | 51 |
| novel_mir_5 | 23 | AAUCUAGUCGUUGAUUUUGGACA | 19 | 0 |
| novel_mir_502 | 23 | UAAAGGGAGGGAGAGUUCGGCUA | 0 | 14 |
| novel_mir_503 | 21 | CCGGAAUUGGAUGUAAGCAGG | 0 | 69 |
| novel_mir_505 | 23 | UCUGGGACGCAUGUGUGGAAACU | 0 | 20 |
| novel_mir_509 | 21 | CAAGCAAGUAGAAGUGCACAU | 0 | 20 |
| novel_mir_510 | 21 | AAGCAAGUAGAAGUGCACAUU | 0 | 20 |
| novel_mir_58 | 21 | AUUGUGGAAGAUGUAGAGUAG | 5 | 15 |
| novel_mir_59 | 23 | AUUUGGAUGGAUUGUUGUAGGUA | 80 | 63 |
| novel_mir_64 | 23 | GUUGAAUUUUGAUCAAACGGUUA | 248 | 214 |
| novel_mir_71 | 21 | UAAUUGACUGUGAAAUCGUGG | 22 | 42 |
| novel_mir_75 | 21 | CAAGGGAUUGAUGUCAAGUUA | 25 | 36 |
| novel_mir_77 | 21 | CUUUCAGGAGAUGUAUUGACC | 9 | 15 |
| novel_mir_80 | 21 | CCUUUGAUGGGAGAGAUCUGG | 130 | 0 |
| novel_mir_82 | 21 | UUAACUUGGCUAUUGUAGAAC | 25 | 8 |
| novel_mir_84 | 23 | CUCUUGACUGUUAGAUUUGGCUU | 25 | 0 |
| novel_mir_88 | 23 | GGGCGUGUGAGAAUGUGAAGGUA | 36 | 0 |
| novel_mir_89 | 21 | UCCACACAUCAUCUAGGACAA | 128 | 111 |
| novel_mir_96 | 21 | UCCAUGAGCAUGACAAAGAUA | 11 | 15 |
| novel_mir_99 | 21 | UGUAGGAGAAAUGGAAUUGGC | 25 | 33 |
