# Supplementary material for: Genome-wide identification of vegetative phase transition-associated microRNAs and target predictions using degradome sequencing in Malus hupehensis
Source: BMC Genomics. 2014 Dec 17;15(1):1125. doi: 10.1186/1471-2164-15-1125 (PMC4523022; doi:10.1186/1471-2164-15-1125)
Supplement: Supplementary file 12 — Additional file 12: Hierarchical clustering of known miRNAs (A) and targets (B) by expression levels in adult and juvenile leaves of Malus hupehensis . Samples are reported on the top side of the heat map with the following codes: Date (from March to August). A: Adult phase leaves from the tree top; J: Juvenile phase leaves from the tree base. (DOCX 131 KB) [file 12864_2014_7075_MOESM12_ESM.docx]

**Additional file 12**. Hierarchical clustering of known miRNAs (A) and targets (B) by expression levels in adult and juvenile leaves of *Malus hupehensis*. Samples are reported on the top side of the heat map with the following codes: Date (from March to August). A: Adult phase leaves from the tree top; J: Juvenile phase leaves from the tree base.

**A**

**Date**

**
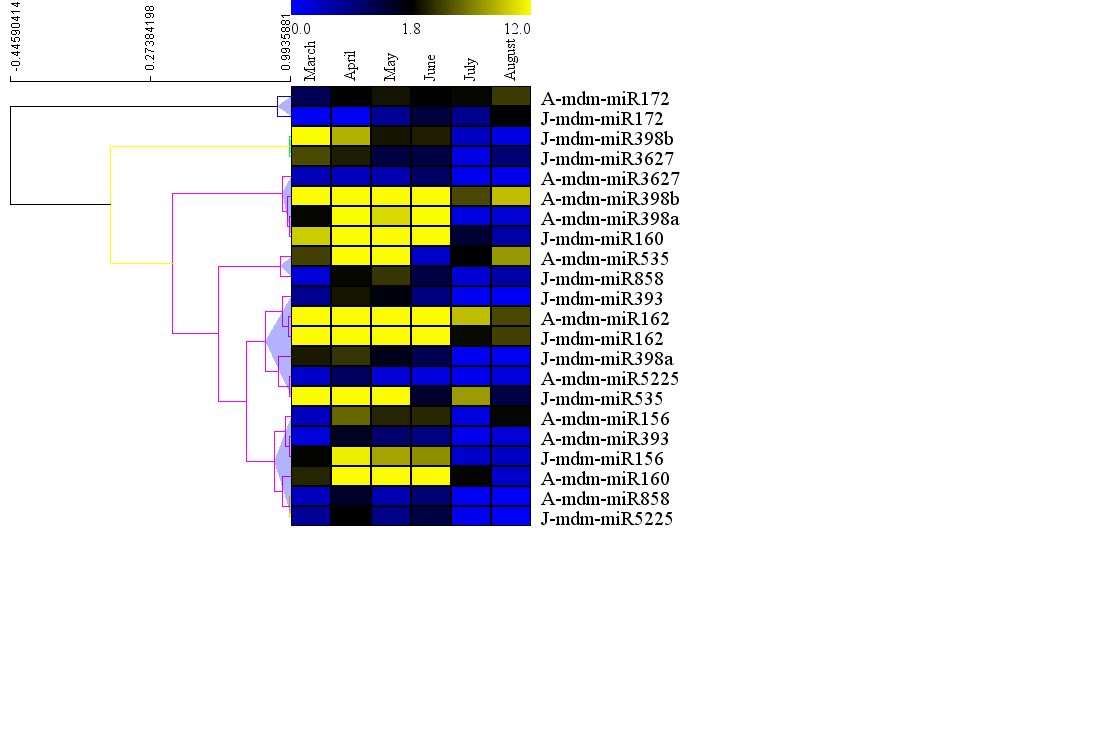
**

**5**

**6**

**4**

**3**

**1**

**2**

**B**

**Date**

**
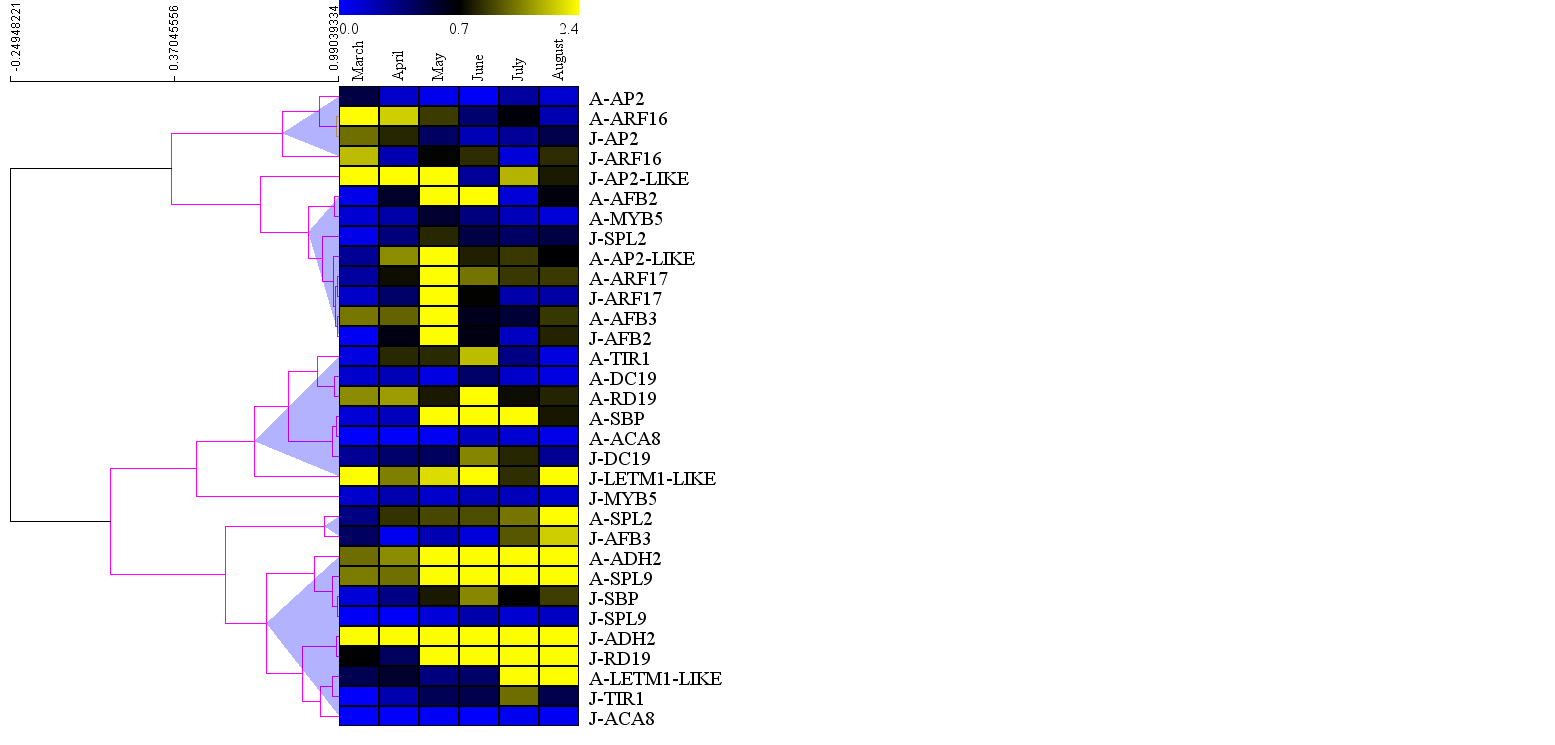
**

**3**

**5**

**4**

**2**

**1**
