# Supplementary material for: Genome-wide identification of vegetative phase transition-associated microRNAs and target predictions using degradome sequencing in Malus hupehensis
Source: BMC Genomics. 2014 Dec 17;15(1):1125. doi: 10.1186/1471-2164-15-1125 (PMC4523022; doi:10.1186/1471-2164-15-1125)
Supplement: Supplementary file 13 — Additional file 13: Hierarchical clustering of known miRNAs (A) and targets (B) by expression levels in Malus hupehensis leaves of different ages. Samples are reported on the top side of the heat map with the following codes: Age (from 1 to 6 years). A: Adult phase leaves from the tree top; J: Juvenile phase leaves from the tree base. (DOCX 75 KB) [file 12864_2014_7075_MOESM13_ESM.docx]

**Additional file 13**. Hierarchical clustering of known miRNAs (A) and targets (B) by expression levels in *Malus hupehensis* leaves of different ages. Samples are reported on the top side of the heat map with the following codes: Age (from 1 to 6 years). A: Adult phase leaves from the tree top; J: Juvenile phase leaves from the tree base.

**Age**


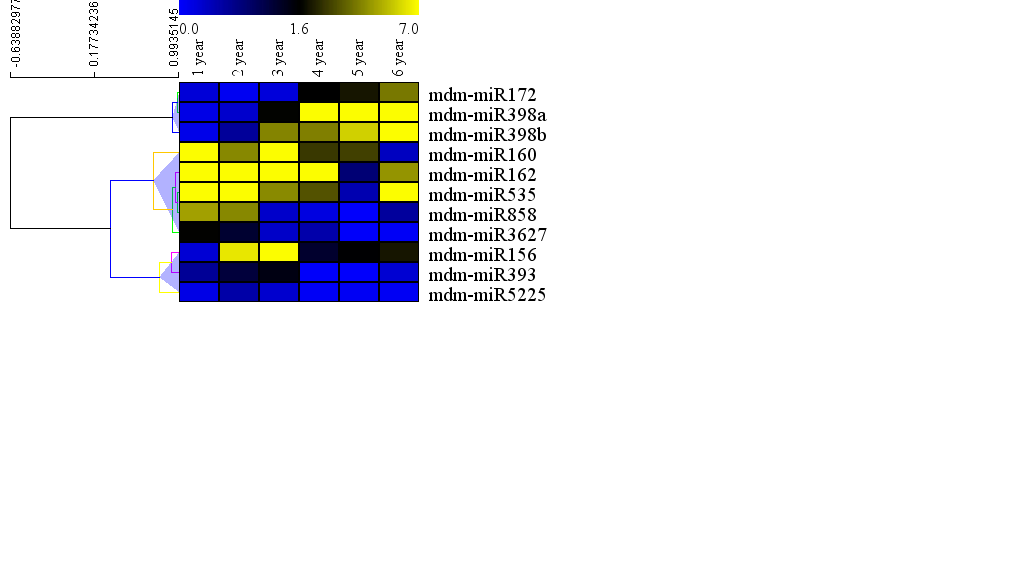


**Age**

**1**

**3**

**2**

**B**

**A**


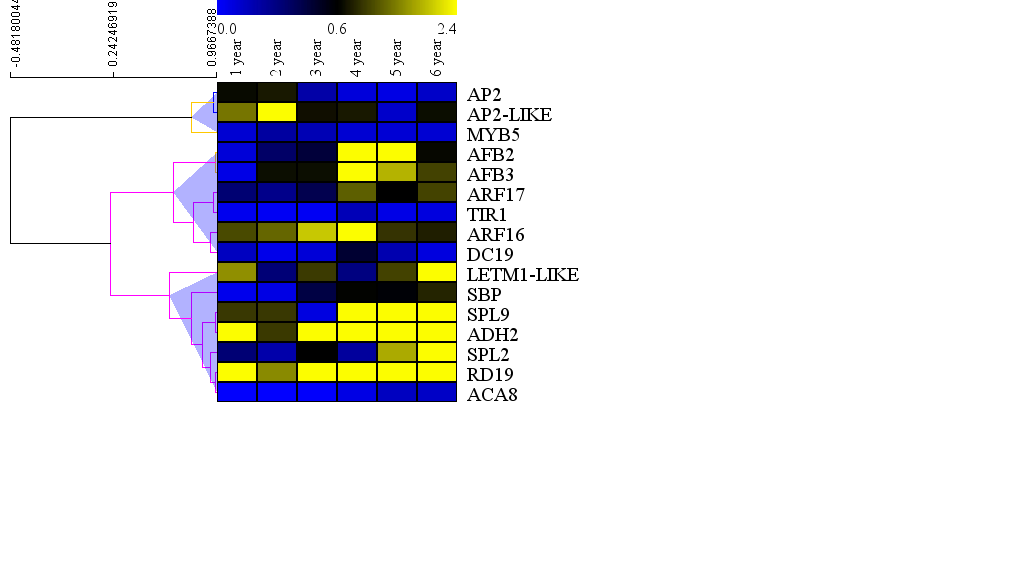


**3**

**2**

**1**
