# Supplementary material for: Genome-wide identification of vegetative phase transition-associated microRNAs and target predictions using degradome sequencing in Malus hupehensis
Source: BMC Genomics. 2014 Dec 17;15(1):1125. doi: 10.1186/1471-2164-15-1125 (PMC4523022; doi:10.1186/1471-2164-15-1125)
Supplement: Supplementary file 14 — Additional file 14: Hierarchical clustering of known miRNAs (A) and targets (B) by expression levels in different Malus hupehensis ’ tissues. Samples are reported on the top side of the heat map with the following codes: Tissues (root, stem, flower, leaf and fruit). A: Adult phase leaves from the tree top; J: Juvenile phase leaves from the tree base. (DOCX 78 KB) [file 12864_2014_7075_MOESM14_ESM.docx]

**Additional file 14**. Hierarchical clustering of known miRNAs (A) and targets (B) by expression levels in different *Malus hupehensis*’ tissues. Samples are reported on the top side of the heat map with the following codes: Tissues (root, stem, flower, leaf and fruit). A: Adult phase leaves from the tree top; J: Juvenile phase leaves from the tree base

**A**


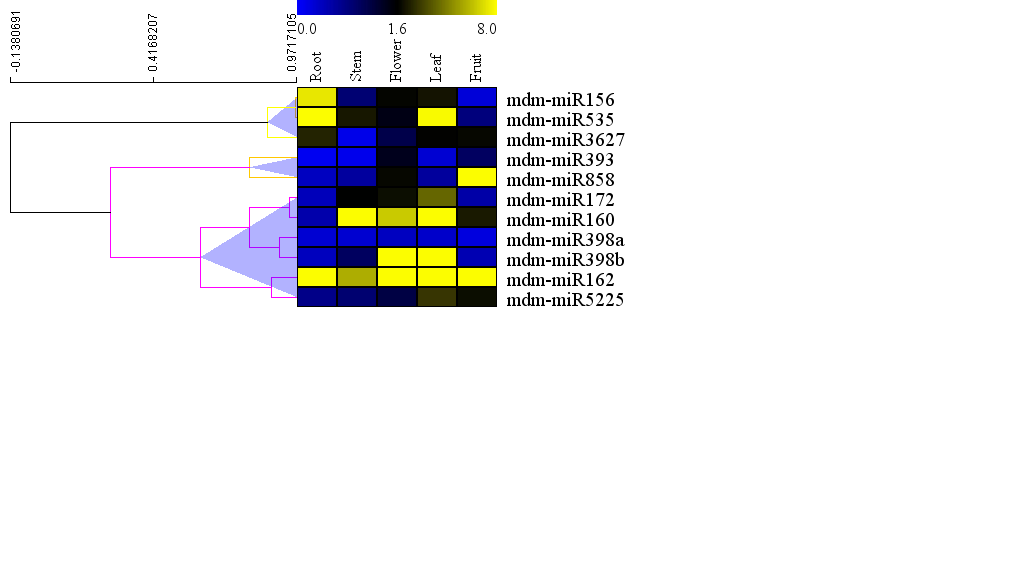


**3**

**2**

**1**

**B**

**Tissue**

**Tissue**


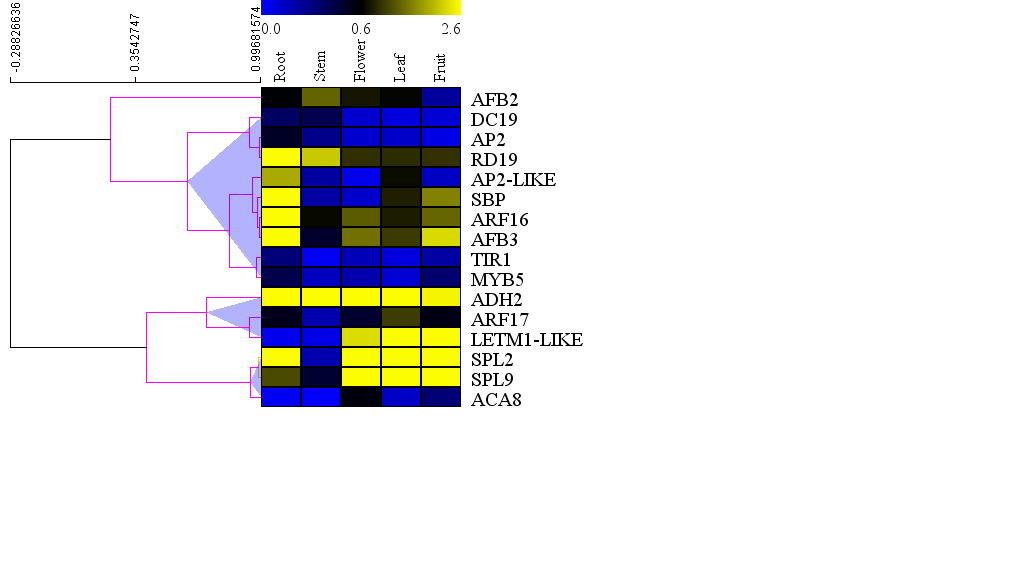


**3**

**2**

**1**
