# Supplementary material for: Genome-wide identification of vegetative phase transition-associated microRNAs and target predictions using degradome sequencing in Malus hupehensis
Source: BMC Genomics. 2014 Dec 17;15(1):1125. doi: 10.1186/1471-2164-15-1125 (PMC4523022; doi:10.1186/1471-2164-15-1125)
Supplement: Supplementary file 15 — Additional file 15: Hierarchical clustering of novel miRNAs with expression levels in adult and juvenile leaves of Malus hupehensis (A) in leaves of different ages (B) and in different tissues (C). Samples are reported on the top side of the heat map with the following codes: Date (From March to August) (A); Age (from 1 to 6 years) (B); Tissues (root, stem, flower, leaf and fruit) (c). A: Adult phase leaves from the tree top; J: Juvenile phase leaves from the tree base. (DOCX 83 KB) [file 12864_2014_7075_MOESM15_ESM.docx]

**Additional file 15.** Identification by qRT-PCR of novel miRNA expression patterns in adult and juvenile leaves of Malus hupehensis (A) in leaves of different ages (B) and in different tissues (C). A: Adult phase leaves from the tree top; J: Juvenile phase leaves from the tree base.
