# Supplementary material for: Genome-wide identification of vegetative phase transition-associated microRNAs and target predictions using degradome sequencing in Malus hupehensis
Source: BMC Genomics. 2014 Dec 17;15(1):1125. doi: 10.1186/1471-2164-15-1125 (PMC4523022; doi:10.1186/1471-2164-15-1125)

**Additional file 16**. Hierarchical clustering of novel miRNAs with expression levels in adult and juvenile leaves of *Malus hupehensis* (A) in leaves of different ages (B) and in different tissues (C). Samples are reported on the top side of the heat map with the following codes: Date (From March to August) (A); Age (from 1 to 6 years) (B); Tissues (root, stem, flower, leaf and fruit) (c). A: Adult phase leaves from the tree top; J: Juvenile phase leaves from the tree base.

**Date**

**A**


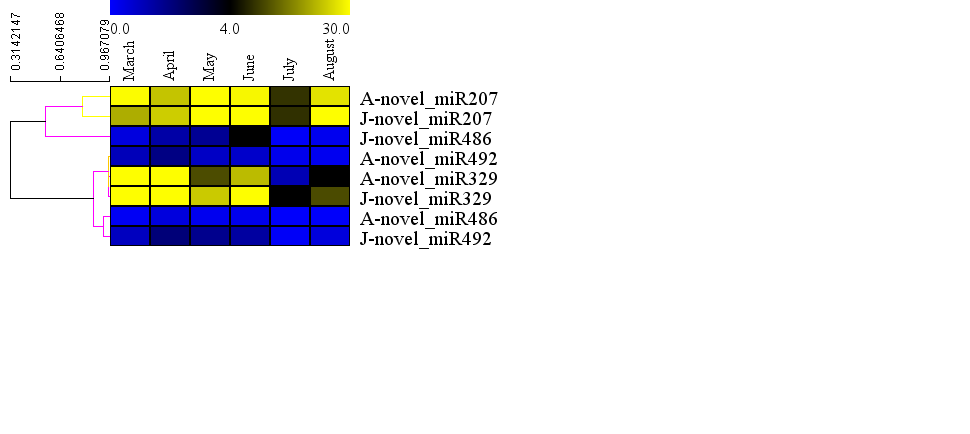


**B**

**Age**


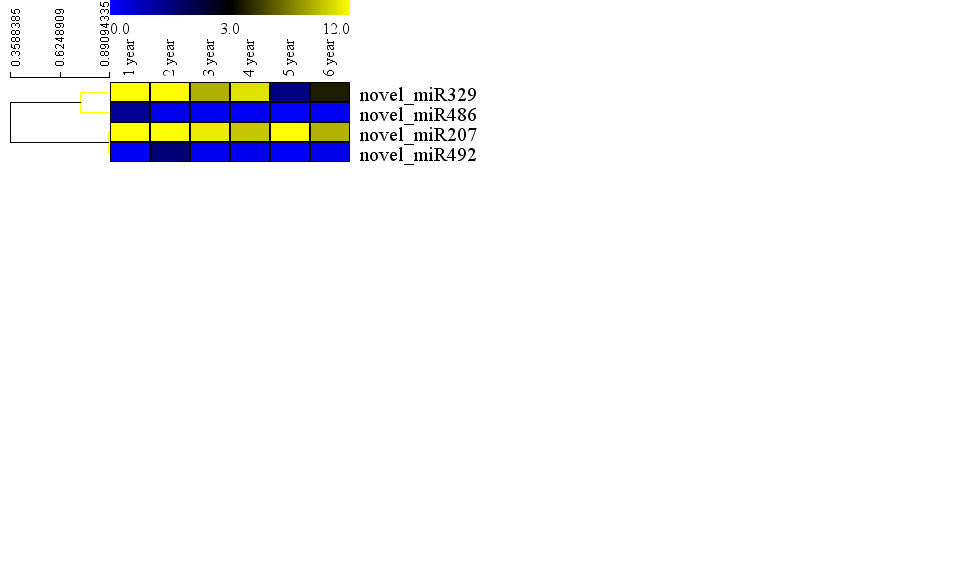


**C**

**Tissue**


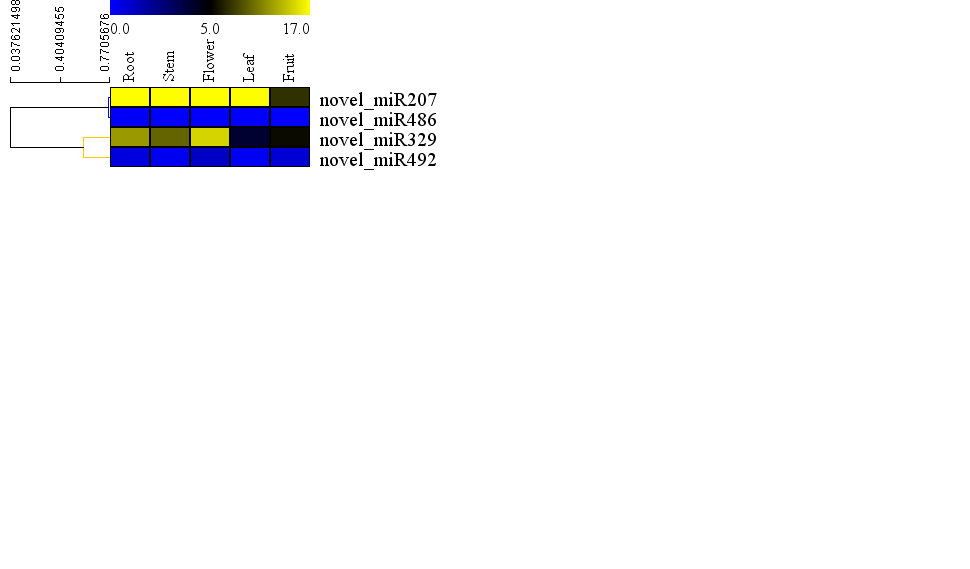

Supplement: Supplementary file 16 — Additional file 16: Identification by qRT-PCR of novel miRNA expression patterns in adult and juvenile leaves of Malus hupehensis (A) in leaves of different ages (B) and in different tissues (C). A: Adult phase leaves from the tree top; J: Juvenile phase leaves from the tree base. (DOCX 75 KB) [file 12864_2014_7075_MOESM16_ESM.docx]
